# Biocultural diversity and crop improvement

**DOI:** 10.1042/ETLS20230067

**Published:** 2023-12-12

**Authors:** Paul Gepts

**Affiliations:** Department of Plant Sciences, Section of Crop and Ecosystem Sciences, University of California, Davis, CA 95616-8780, U.S.A.

**Keywords:** agricultural biodiversity, biodiversity, crop improvement, gene flow, polyploidy, traditional knowledge

## Abstract

Biocultural diversity is the ever-evolving and irreplaceable sum total of all living organisms inhabiting the Earth. It plays a significant role in sustainable productivity and ecosystem services that benefit humanity and is closely allied with human cultural diversity. Despite its essentiality, biodiversity is seriously threatened by the insatiable and inequitable human exploitation of the Earth's resources. One of the benefits of biodiversity is its utilization in crop improvement, including cropping improvement (agronomic cultivation practices) and genetic improvement (plant breeding). Crop improvement has tended to decrease agricultural biodiversity since the origins of agriculture, but awareness of this situation can reverse this negative trend. Cropping improvement can strive to use more diverse cultivars and a broader complement of crops on farms and in landscapes. It can also focus on underutilized crops, including legumes. Genetic improvement can access a broader range of biodiversity sources and, with the assistance of modern breeding tools like genomics, can facilitate the introduction of additional characteristics that improve yield, mitigate environmental stresses, and restore, at least partially, lost crop biodiversity. The current legal framework covering biodiversity includes national intellectual property and international treaty instruments, which have tended to limit access and innovation to biodiversity. A global system of access and benefit sharing, encompassing digital sequence information, would benefit humanity but remains an elusive goal. The Kunming-Montréal Global Biodiversity Framework sets forth an ambitious set of targets and goals to be accomplished by 2030 and 2050, respectively, to protect and restore biocultural diversity, including agrobiodiversity.

## Introduction

### Biodiversity is an exquisite and irreplaceable environmental resource on Earth

The most distinctive feature of Planet Earth compared with other known celestial bodies is the vibrant, fascinating, and ever-evolving biological ‘tapestry’ at or near its surface, biodiversity, which consists of all ‘plants, animals, fungi, and micro-organisms, their genotypic and phenotypic variation, and the [populations,] communities, and (agro)ecosystems of which they are a part’[1,2]. This biological diversity (hence, biodiversity) is present in a wide range of terrestrial (including soils), air, and marine environments, from the frozen tundra near the Poles to the steamy jungles near the Equator and from the driest deserts to rain-drenched forests. Although tropical ecosystems include the largest number of species, living organisms have penetrated and adapted to all possible habitats on Earth. The current status of this biodiversity is the result of 600 million years of evolution (the Phanerozoic Period) on the Earth and its continuous natural cycle of species appearance and extinction as a function of the complex yet subtle interactions within ecosystems, landscapes, and biomes among the species they include and the genetic building blocks that constitute these species.

Biodiversity can be assessed at three hierarchical and complementary levels: (1) individual or population, (2) species (existing or extinct), and (3) ecosystem. There are some ∼8.7 million species, of which only 14% of land species and 9% of ocean species have been described taxonomically. Given the current extinction rates, many species may become extinct before they are ever described by science [3]. Among plants, it has been estimated that there are some 400 000 species, of which 10–20% are unknown [4–6]. Areas rich in vascular plant species include the Americas (from Mexico to Bolivia; and in central and southeastern Brazil), Asia (from southern China, Vietnam, and Thailand, to the Southeastern Asia and New Guinea islands), Australia (especially Western Australia, Queensland and New South Wales), Africa (coastal Gabon and Cameroon, the Albertine Rift, the Eastern Arc Mountains in Kenya and Tanzania, and the Drakensberg and Cape Region of South Africa), and Madagascar [7]. Regions with the highest number of plant species all originated within the Tropics and Subtropical Moist Broadleaf Forest vegetation type [8]. An extensive sample of 320 000 species showed that tropical and subtropical islands, as well as tropical mountain regions, encompassed the highest level of endemism (geographic uniqueness), reflecting environmental heterogeneity, long-term climatic stability, and geographic isolation [9]. Attempts at synthesizing the Earth's biodiversity has led to the establishment of the Tree of Life [10], the Earth Biogenome Project [11], and the World Flora Online (http://www.worldfloraonline.org/). Many but not all these areas overlap with centers of agricultural origins [12–14].

Biodiversity provides several crucial roles that benefit humanity, including increased biological productivity and stability, dietary diversity, tolerance to biotic and abiotic stresses, and enhanced ecosystem services ([15]; see also Functions and uses of biodiversity). It is, therefore, a paradox that, despite its pervasive and profound utility, biodiversity suffers increasingly from deleterious human actions ([2,16–18]; see also Trends in crop biodiversity), leading to a massive rate of extinction among plants and animals especially in high-diversity regions with a tropical or Mediterranean climate, including islands [16,19]. Even though plants make up ∼80% of the total biomass on the planet and provide part of the oxygen in the atmosphere (in addition to algae and photosynthesizing bacteria), play a crucial role in ecosystem functions, and support humans by delivering multiple benefits [20], the awareness of the importance of plants and the need for their conservation have taken a backseat to animal initiatives [21]. The proportion of assessed vascular plant species that are considered threatened varies between datasets: 37% (ThreatSearch) and 44% (International Union for Conservation of Nature Red List of Threatened Species) [22]. Among undescribed plant species, three in four species face extinction [23]. Most extinct plants are woody perennials or plants from the wet subtropics or tropics [19]. There is seemingly no phylogenetic pattern to the risk of extinction, as all but two of the nine plant phylogenetic lineages harbor more losers than winners [19,24].

### The joint biodiversity loss — climate change crisis

A close link exists between the two major environmental crises affecting our planet: biodiversity loss and climate change [25]. The latter will undoubtedly lead to a hotter world due to the untrammeled emission of greenhouse gases such as CO_2_, especially since the Industrial Revolution in the 19th century. While higher CO_2_ levels stimulate photosynthesis, especially in C3 plants, other climate change effects negate the potential benefits of CO_2_, such as rapidly increasing average temperatures. Even if greenhouse gas emissions were to be halted now, the composition of the atmosphere, especially in the content of CO_2_, the most abundant greenhouse gas, will decrease only slowly after that, and global temperatures will remain above pre-industrial levels for the foreseeable future. Only plant species that tolerate and adapt to human pressures and shift distributions will be winners and survive the Holocene or Anthropocene extinction, the 6th mass extinction, but one for which humans are directly responsible [26]. The shift in distributions is dependent on the presence of seed dispersers, themselves affected by biodiversity loss [27]. Because most plant (and fungal) species risk assessments do not yet capture the full range of threats like global climate change or the combination of threats, plants as a whole are moving toward extinction, a trend hidden by the lag phase plant species show in revealing threats to their existence due to their adaptation to local environments [22].

The bleak future for plants is illustrated by the observation that ∼29°C mean annual temperature, the productivity of five lowland tropical trees from Central America declined sharply (−11% per 1°C increase). The productivity of dry tropical reforestation nearly halved under a high-emission scenario [28]. In the Sierra Nevada of California, low-elevation conifers are out of equilibrium with the current climate leading to a vegetation-climate mismatch (VCM), potentially resulting in vegetation conversions. About 20% of Sierra Nevada coniferous forests suffer from a VCM, mainly at low altitudes [29]. Although the global mean number of days above freezing will increase by up to 7% by 2100 (under the «business as usual» RCP 8.5 greenhouse gas emission scenario), suitable growing days will decrease globally by up to 11% when considering other climatic variable like water availability and solar radiation. Tropical areas will become too hot and lose up to 200 growing days per year. Under strong (RCP 2.6) and moderate (RCP 4.5) mitigation scenarios, changes in suitable growing days for plants will be less severe but will nevertheless cause severe human socio-economic distress and injustice because of reduced access to plant-based products and services [30]. Such situations are already leading to migration crises in various parts of the world [31,32]. Thus, global warming and climate change constitute a joint crisis [33], in which the two threats are operating in concert but could also jointly provide mitigating solutions.

### The domestication triangle as a reflection of crop biocultural diversity

Given the multiple roles exercised by biodiversity, it should come as no surprise that biodiversity also has strong links to the socio-economic conditions of our societies and is referred to as biocultural diversity [34–36]. Examples abound of the multiple connections between plant biodiversity and cultural diversity. These include traditional knowledge and environmental ethics of indigenous societies (e.g. [37–39]), the linguistic diversity related to biodiversity identifications [40–42], the gastronomic diversity linked to plant ingredients (e.g. [43,44]), the diversity of the cultivated stock of individual countries (e.g. India [45]), gene flow among wild, weedy, and domesticated plant types (e.g. potato [46]), and the role of traditional agroecosystems in assuring food self-sufficiency (e.g. Mexico: milpa [47], agri-silviculture [48]). Nevertheless, in wealthy societies but to a lesser extent in the Global South, plants suffer from a lack of awareness, as alluded to earlier [49,50]. This has long-term consequences for our survival as a species, the foundations of our general knowledge, the existence of industries, and our overall well-being.

**Figure 1.**
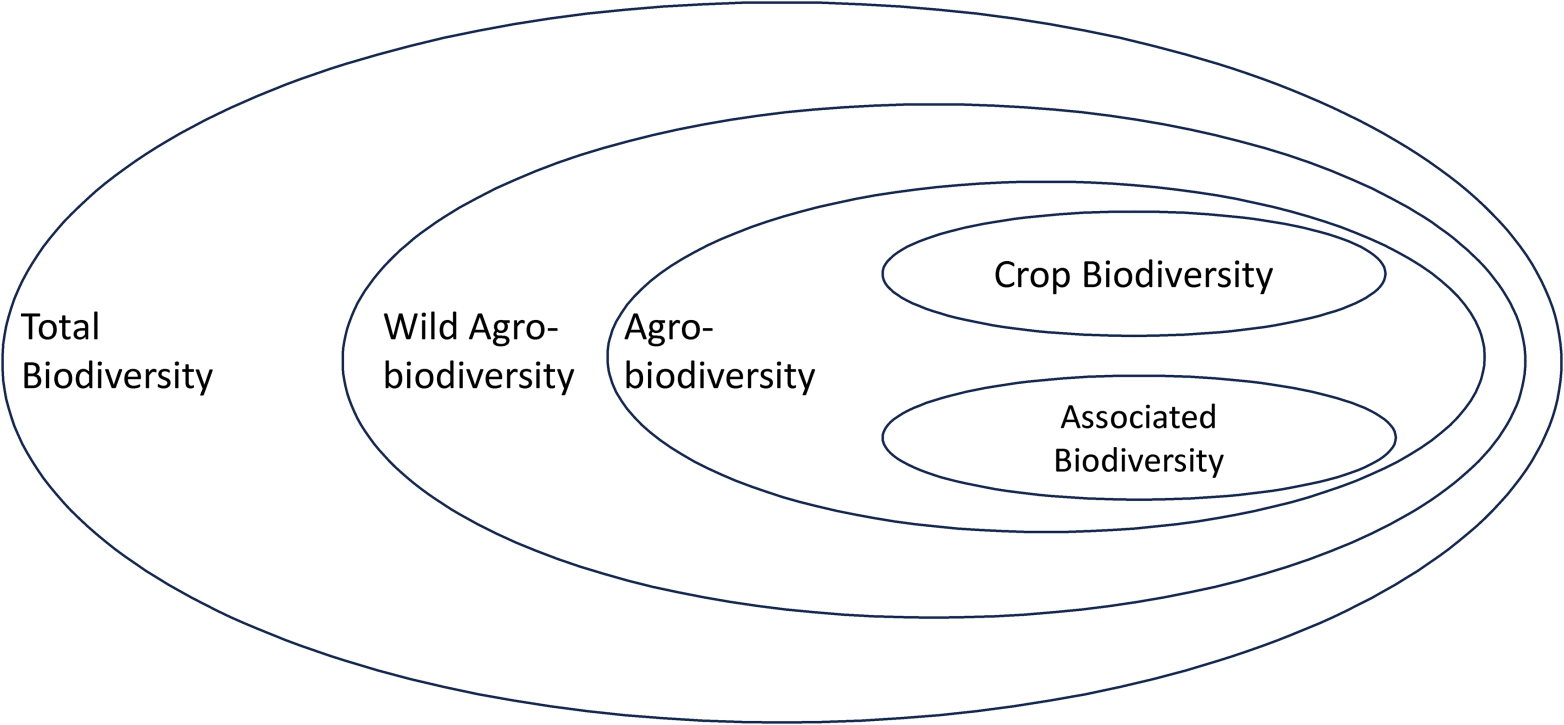
Agrobiodiversity is an economically important subset of the total biodiversity on Earth. The nested sets represent different gene pools, but are not drawn to scale, i.e. it do not reflect the number of species or other measures of genetic diversity. The wild agrobiodiversity pool includes the wild relatives of crop plants and other biodiversity (pollinators, micro-organisms) in the immediate surroundings of cultivated fields (hedges, wall, etc.) or within pollinating distances. The agrobiodiversity pool represents the biodiversity present in fields, orchards, or forests, including the biodiversity of crops but also the diversity of associated organisms from micro-organisms (e.g. rhizosphere, phyllosphere, endosymbionts) to pollinators.

**Figure 2.**
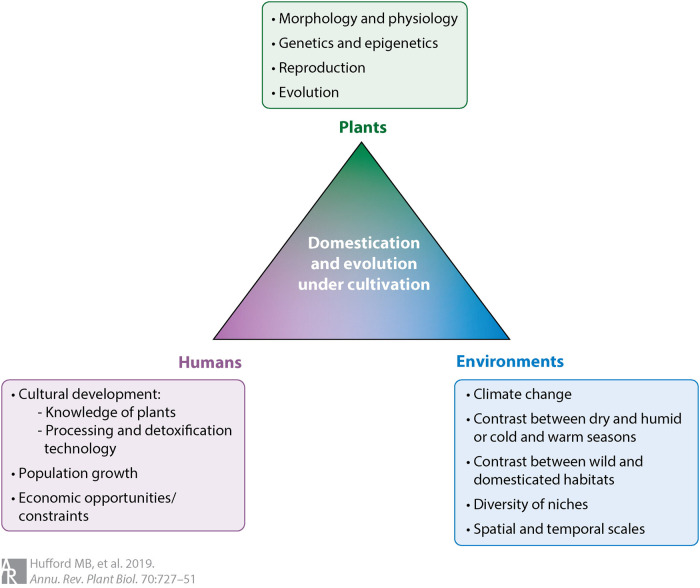
Domestication and subsequent crop evolution are major factors in crop biodiversity, which, in turn, depends on the interaction of plant, environmental, and human factors. The ‘Domestication Triangle’ figure lists examples of specific factors that have been implicated in domestication and subsequent evolution ([52].

The biodiversity of direct relevance to crop improvement is a small but economically and nutritionally highly relevant subset of the Earth's total biodiversity — agrobiodiversity (Figure 1) — which arose initially from domestication during the transition from hunting-gathering to agriculture some 12 000–10 000 years ago. The ‘domestication triangle’ (Figure 2) includes the three major factors that affect the process of domestication and, therefore, domesticated or crop biodiversity: (1) the biological characteristics of the organism, (2) the biotic and abiotic environmental characteristics, and (3) human influence [51,52]. The domestication triangle concept also applies to contemporary (e.g. [53]) and potential future situations affecting crop biodiversity and, therefore, our food systems (e.g. [54]).

Agrobiodiversity also includes plant interactions with other non-plant organisms [55], such as symbionts [56,57], pollinators [58–60], soil organisms [61], and plant microbiomes (e.g. phyllosphere [62]; rhizosphere [63]). In this context, plants — in general — fulfill numerous functions and provide a wide range of amenities to humans. Plants clothe, feed, house, cure, and entertain us [20]. Some 80% of our food derives from plants, according to the Food and Agriculture Organization of the U.N. Close to half of the people on Earth rely mainly on natural medicines, and some 70% of cancer drugs are derived from plants or are plant mimics. More than three-quarters of crops, including fruits and vegetables and cash crops like coffee, cacao, and almonds, are pollinated by animals, primarily insects [17]. Regarding crop biodiversity, considerations regarding the geographic and ecological distribution of domesticated biodiversity, the ways of measuring this biodiversity, its multiple functions, and its conservation are similar to those of natural biodiversity but with changes of emphasis towards population and species diversity.

### Crop biodiversity is conserved *in situ* and *ex situ*

Biodiversity, in general, and crop biodiversity, specifically, can be conserved on-site (*in situ*) and off-site (*ex situ*) [64]. On-site conservation includes mainly farms where crops are cultivated [36,65] and natural vegetation where wild plants grow [66–68]. In contrast, off-site conservation involves an extensive network of gene banks where collections of crop genetic diversity are maintained. These *ex situ* collections are based on propagative materials, such as grains for seed-propagated crops, orchards, or tissue-culture-based collections for vegetatively propagated crops [68–70].

Countries generally have national gene banks, but there are also international gene banks like those operated by the Consultative Group on International Agricultural Research in its network of research centers [68]. Citizens initiatives complement these governmental gene banks, with a more focused target, such as Native Seeds (https://www.nativeseeds.org/) [71] focused on the U.S. Southwest, the Seed Savers Exchange focused on heirloom varieties of food crops (https://seedsavers.org/), seed libraries (e.g. [72]) and community seed banks (e.g. [73]). A global backup for many gene banks is the Svalbard Global Seed Vault, operated as a partnership between the Ministry of Agriculture and Food of the Government of Norway, the Nordic Genetic Resource Center (NordGen), and the Global Crop Diversity Trust (https://www.croptrust.org/).

*Ex situ* and *in situ* conservation complement each other as they have advantages and disadvantages [74,75]. Despite the importance of these conservation approaches, they tend to be underfunded. Their utilization is faced with several bottlenecks, including the lack of genotypic and phenotypic characterization and evaluation, the scarcity of available information on individual entries, and the need to conduct pre-breeding as a first step in the breeding process to adapt the gene bank accessions to local breeding conditions. The availability of databases that combine different types of information related to each accession is a long-awaited goal [76]; components of a future, more integrated information system do exist, such as GERMINATE [77]. For example, the Genesys-PGR database (https://www.genesys-pgr.org) is an online aggregator portal grouping information about holdings of gene banks worldwide. Other platforms are EURISCO (http://eurisco.ecpgr.org, [78]) and GRIN-Global (https://npgsweb.ars-grin.gov/gringlobal/search). These portals contain primarily passport data [taxonomy, biological status (domesticated, wild, weed), basic phenotypic data (e.g. seed size, color, days to flowering), collecting origin], and some phenotypic evaluation data. In contrast, other databases are focused on the genetics and genomics of groups of plants, such as the Legume Information System (LIS, the Legume Information System: https://www.legumeinfo.org/) and Gramene (mainly cereals: https://www.gramene.org/). The works cited in this section and the next ones provide several examples of phenotypic and genotypic characterization studies.

To enhance the utilization of genetic resources collections, the National Genetic Resources Advisory Council (NGRAC) of the U.S. Department of Agriculture recommended an increased emphasis on the evaluation (genotypically and phenotypically) and genetic enhancement (pre-breeding; see The use of biodiversity in genetic improvement). To facilitate this evolution in emphasis, the NGRAC also recommended partnerships and targeted grant programs with institutions outside the U.S.D.A. and further development of the functionalities of GRIN-Global (https://tinyurl.com/ysorhbe8).

## Functions and uses of biodiversity

### Biodiversity — productivity relationship

There is generally a positive relationship between biodiversity (whether wild or cultivated) and productivity, whether this relationship is assessed at the level of a population or field or an ecosystem and regardless of vegetation types (e.g. grassland [79,80]; forage mixtures [81];forests [82]; agroforestry [83–85], see The use of biodiversity in cropping improvement); and silvo-pastoral systems [86]). In a long-term (11-year) study of a native grassland and three successional grasslands in the northern U.S., more diverse plant communities were more resistant to a drought stress that led to a >45% reduction in aerial living biomass [79]. In addition, these diverse communities recovered more completely from the same stress. The concave-downward curve showing the relationship between plant species richness and biomass suggested that each additional lost species had a gradually larger impact on drought susceptibility; conversely, species richness led to greater drought tolerance because species-rich plots had a greater probability of containing some drought-tolerant species, which could compensate partially for the drought susceptibility of other species. This same study also showed that species-rich plots recovered their pre-drought biomass in contrast with species-scant plots, which were more damaged by drought stress and took longer to recover. The authors attributed the tolerance and resilience to drought to the sheer diversity (number of species). Still, one could also suggest that the variety of drought tolerance functionalities would increase in species-rich vegetations.

This was investigated further by comparing the effect of the number of plant species and functional groups and the functional plant composition in individual plots [80]. Among functional groups, legumes vs. grasses, C3 vs. C4 grasses, and woody plants vs. forbs were considered. The functional groups and composition components of diversity were more significant factors in ecosystem processes than the species component. Hence, ecosystem modifications that affect composition, such as invasive species, disturbances, fragmentation, and management practices, likely affect ecosystem processes [80].

Forests are significant because they harbor a significant proportion of terrestrial biodiversity. In a study [82] of the effect of tree species richness on tree volume productivity across most of the global terrestrial biomes, encompassing some 8700 species, a positive concave-down effect was observed of tree biodiversity on forest productivity, indicating that biodiversity loss would also negatively affect the production of forest products. The effect was stronger percentage-wise in boreal regions but higher in absolute terms in tropical forests. This analysis highlights the potential benefits of mixed-species forest management compared with tree monocultures. Another aspect is the ancillary biodiversity, including herbaceous and ligneous non-timber species in these forested biomes. These species were not included in this study but may also provide benefits or ecosystem services and suffer losses in tree monocultures.

The positive relationship between biodiversity and productivity also holds in complex, non-experimental landscapes that provide ecosystem services to humans. Landscape-scale productivity and stability increased with plant and other biodiversity over large environmental and altitudinal gradients. The effects of biodiversity on productivity were similar in magnitude to those of other drivers like climate, topography, and land cover [87].

A meta-analysis of 45 studies [88] examined whether plant diversification reduced herbivore (insect pest) impacts (or increased the role of natural enemies of herbivores). They reported that herbivore suppression, enemy enhancement, and crop damage reduction were higher on diversified cropping systems than on monocultures with no or few associated plant species. There was also a modest reduction in yield, which the authors attributed to the partial replacement of the main crop with associated crops or non-crop plants. Overall, these results were consistent with an increase in biological productivity associated with increased diversity and encouraged further research into plant diversity as an alternative to pesticide-driven industrial monocultures.

### Biodiversity — sustainability relationship

The relationship between biodiversity and productivity has taken on ever more significance because of the conjunction of several trends that lead to a severe imbalance between supply and demand for food, feed, timber, and other biological products. These trends include a relentless increase in the size of the human population [89], the emission of greenhouse gases from various sources, including agriculture, which accounts for 30% of the total [90], and the destruction due to multiple causes of terrestrial and marine habitats [91], despite the repeated warnings of scientists and calls to action by civil society and citizens over several decades [92–94].

Whereas biodiversity is one of the «victims» of the perfect storm affecting our planet at the hands of *Homo sapiens*, it is also proposed as a partial solution to this storm. There are at least some 7000 edible plant species [95,96], most of which have additional uses, such as medicinal (70%), materials (60%) and environmental uses (40%). This large number of edible species contrasts with the limited number of food species relied on for actual global production and consumption [97]. Furthermore, the downward diversity trend results in an increasing focus on carbohydrate and oil crops, with adverse effects on our diet and chronic disease status [98]. This situation led to the suggestion of an increased focus on neglected and underutilized species (NUS), which include wild, semi-domesticated, and domesticated plants that can potentially improve people's food security and livelihoods because they are locally significant to people and often adapted to unique climatic and environmental conditions [95,99].

It has also been proposed to augment the number of crop species cultivated in a country and, hence, the national crop diversity to decrease crop losses to stresses, notably climate events, especially droughts, which cause 50% of unexpected drops in national food production (e.g. maize in the U.S.) and, conversely, increase year-to-year stability. Crop species diversity had the greatest stabilizing effect, followed by irrigation; fertilization had no effect. The national crop portfolio should also focus on nutritional diversity to better fulfill dietary diversity, primarily based on plant-based foods [100]. Similar proposals [101,102] also focused on crop diversity but at the ecosystem and population (plant breeding) levels (see The use of biodiversity in cropping improvement and The use of biodiversity in genetic improvement).

### Biodiversity — ecosystem services relationship

Ecosystem services can be defined as tangible or intangible, direct or indirect contributions of ecosystems to human well-being. There are four broad categories of services [103], including provisioning (e.g. food, raw materials, fresh water, medicines), regulating (e.g. flood regulation, water purification, and pollination), cultural (non-material benefits, e.g. spiritual enrichment, recreation, and esthetic values), and supporting services (e.g. water cycle, photosynthesis; soil formation, habitat for species; and genetic diversity between and within population). Biodiversity plays a role in each of these four types of services and, hence, needs to be maintained or enhanced if human societies are to continue benefiting from these services.

How can agrobiodiversity be enhanced in agricultural ecosystems while achieving production goals like increased yield and improving ecosystem services? Agriculture in the U.S. Corn Belt is dominated by a near-monoculture of corn-soybean rotation strongly dependent on petrochemical inputs. Environmental issues include water contamination by nutrients and pesticides from croplands and a dearth of habitats supporting native plants and animals, including pollinators (e.g. [104]). Cropping system research [105] showed that (1) the addition of small grains and forage legumes to the corn-soybean rotation allowed for marked reductions in petrochemical or fossil fuel use with reducing yields or profitability; and (2) intercalation of prairie buffer strips consisting of native plants provided significant improvements in soil and water conservation and nutrient retention.

In a set of 12 multiyear experiments [106], anthropogenic environmental changes affected ecosystem service stability primarily via their effect on biodiversity. Anthropogenic drivers included plant diversity, nitrogen, carbon dioxide, fire, herbivory, and water, each affecting ecosystem productivity. The stability of this productivity was only affected by those drivers affecting biodiversity. In a meta-analysis of diversification practices and their potential effects on crop yield [107], diversification targeting above-ground biodiversity increased pollination, herbivore control, and water regulation, while those targeting below-ground biodiversity improved nutrient cycling, soil fertility, and water regulation while maintaining yields. They cautioned about variability in the actual responses because of context dependency. This observation was further highlighted in a synthesis of some 100 meta-analyses on 120 crop species over 84 experiment years [108]. Crop diversification approaches, such as agroforestry, intercropping, cover crops, crop rotation, or variety mixtures, enhanced crop production (median effect: +14%), but also the associated biodiversity of non-cultivated plants (+24%), water quality (+51%), pest and disease control (+63%), and soil quality (+11%). There were substantial differences among the approaches: agroforestry was the most effective in delivering multiple ecosystem services; in contrast, varietal mixtures provided the fewest benefits. Overall, however, increasing the diversity of crops is an effective strategy for a more sustainable agroecosystem management.

### Biodiversity — socio-economic drivers

Approaches to maintaining or increasing biodiversity in (agro) ecosystems have several benefits for biological and ecosystem services. These approaches take place and have to make sense in a socio-economic context. Two broad land use categories exist to meet rising food demand and maintain biodiversity. In land sharing, the two objectives are pursued on the same land; in land sparing, high-yield farming and protection of natural habitats are kept separate. A comparison of crop yields and biodiversity (measured by the number of bird and tree species) in southwest Ghana and northern India [109] showed that for biodiversity maintenance, land-sparing was a more promising strategy to minimize the negative impacts of agricultural production. However, one should keep in mind that the two land use categories are two extremes along a broad range of options. Notably, agroforestry (see The use of biodiversity in cropping improvement) can achieve the two goals on the same land. In the coming decade (2023–2032), crop production increases will be driven mainly by improvements in productivity (yield) rather than expansion of agricultural land use [110]. It remains to be seen how this trend will benefit biodiversity conservation. To address the issue of land use effects on biodiversity and ecosystem services, a classification of different types of agricultural land managements (e.g. monoculture, crop rotation, crop-livestock, grasslands, and agroforestry) has been proposed into low, medium, and high intensity [111].

As mentioned earlier [100], crop diversity stabilizes national food production. The benefits of this stabilization must be weighed against the damages and expenses incurred in situations destabilizing agricultural production, such as increasing global climate variability. In two-thirds of the cases studied [112], agrobiodiversity (measured mainly as the number of animal and plant species and food products) had a positive effect on food security (including availability, access, and utilization). Yet the magnitude of the relationship between agrobiodiversity and food security is highly variable. It depends on specific agricultural systems, a situation also observed in Tajikistan, Egypt, and the U.S. (state of Arizona) [113].

Agricultural policies also affect biodiversity in agroecosystems. An example is the choices made in research and extension programs regarding the types of biodiversity that will be researched and promoted in extension/outreach programs. As an example [114], economic and social drivers affected the adoption of modern varieties or the continued cultivation of landraces, defined as ‘geographically and ecologically distinct plant populations managed [and improved] by local farmer-breeders’ ([115], brackets by author). They observed a co-existence between landrace cultivation and market-oriented production in the Yunnan rice fields of China. The adoption of the modern HongYang cultivar was strongly determined by urban market demand for this cultivar, whereas seed availability and yield were not substantial factors. The eating habits and taste preferences of urban consumers, the presence of influential farmers, and the existence of seed exchange networks also affect the fate of landraces or cultivars [116–121].

Similar observations about the differential penetration of high-yielding varieties have been made in other countries. In India, breeding programs were activated to develop high-yielding varieties of cereals. However, the adoption of these varieties was unequal. The original landraces continued to be cultivated in different regions or seasons because they were locally adapted or had unique nutritional, medicinal, or therapeutic qualities. Some of these were protected through Plant Variety Protections and Farmers’ Rights legislation ([122]; see also Legal, policy, and valuation framework). The intellectual property regime governing ownership of biological diversity affects the maintenance of existing diversity but also the development of novel diversity by plant breeding or farmer selections [123–125] b and, ultimately, the downstream food system and food security [126].

### Biodiversity — indigenous or traditional knowledge and linguistic diversity

Many centers of crop origin and diversity are located in regions where native or indigenous people play an important role in fashioning population, species, and ecosystem biodiversity. In this context, farmers are not passive recipients of genetic diversity, but through their knowledge, they are aware of existing biodiversity and shape this biodiversity grown on their farms and in the surrounding vegetation. This knowledge and awareness are idiosyncratic but reflect the culinary, dietary, and economic needs of the respective farming enterprises (e.g. common bean: in Mexico [127–129]; in Uganda [120]). The articles cited document the genetic footprint and consequences of farmer selection, including the change in within- vs. among population genetic diversity, the *creolization* of introduced improved cultivars (see also The original process of domestication during the transition from hunting-gathering to agricultural production), and the occasional rigor of farmers in maintaining the phenotypic purity of specific seed types (e.g. [120]).

The peculiarity of traditional knowledge with and between farmer populations can be traced partly to socio-economic factors characterizing these populations and their individuals. In a study of palms (Arecaceae), gender was the only factor at the general palm knowledge level showing a significant association across the five South American regions studied. Otherwise, a highly localized association between socio-economic factors and traditional knowledge was observed, consistent with the idiosyncrasy of this knowledge [130]. Because of the active intervention of individual farmers in selecting specific genetic diversity, farm biodiversity can be considered part of the unique capital of the farm instead of being a mere input as in industrialized agriculture. Smallholder-based agroecology is a win–win outcome [102]. These family farms are responsible for ∼50% of global food production. They tend to produce diverse crops, mainly for local consumption but also for regional and export production; a solid social and natural capital also characterizes them.

What types of knowledge constitute traditional knowledge, and what socio-economic factors influence this knowledge? Traditional societies have relied for millennia on plants for various needs. An example of a historical record is the Códice Florentino, which recorded the different plant uses by the Aztecs. These uses included — in decreasing order of the number of mentions ([131]: Table 5, p. 48) — medicinal, edible, ceremonial, ornamental, industrial, drugs, fuels, construction, forages, dye, fibers, resins, taxation, poisons, spices, sleep-inducing compounds, toys, and glues. Other considerations include agronomic characteristics like growth habit, life cycle, and adaptation. So, biodiversity cannot be considered only as a biological material with the attendant DNA and other biochemical components; also important is the human knowledge about utilizations of biodiversity, hence, the term biocultural diversity [132,133].

As biodiversity goes, so do traditional biological and ecological knowledge. For example, medicinal plants and traditional knowledge in Ethiopia are threatened at a disquieting pace due to habitat destruction, including deforestation, ecological displacement, urbanization, and agricultural expansion [134]. The main factor for the loss of traditional knowledge and agricultural biodiversity in northeast Brazil is the disinterest of younger generations in agriculture. Hence, there is a need to integrate scientific and traditional knowledge to develop sustainable agroecosystems with the assistance of government and private support. One of the foci of the development of sustainable agroecosystems could be on-farm conservation of locally adapted crops such as Lima bean (*Phaseolus lunatus*) [135]. Arid North America (northwest Mexico and southwest U.S.A.) could be used as a potential model for future agriculture under global change towards a hotter and pluviometrically more uncertain climate [136]. They suggested novel arid ecosystems inspired by the Comcaas, O'odham, and Pima Bajo peoples of the Sonoran Desert. These ecosystems were based on a selection of 17 desert food plant genera incorporated into perennial polycultures to simultaneously achieve agriculture resilience, human health, and community prosperity.

Conserving biocultural diversity creates its challenges in addition to those focused on biodiversity *per se*. First, it requires awareness of indigenous science and integration of this science with academic science [137]. Indigenous scientific knowledge complements academic science because it is place-based and assembled over centuries and millennia, e.g. species distributions, habitat needs, and uses. Conservation approaches based on the combination of indigenous and academic sciences are likely more robust and more holistic than either alone. Second, this knowledge is often conveyed in local languages other than English, the current scientific lingua franca: e.g. Albanian [138]; Mexico: Spanish and Mesoamerican languages [139]; Brazil: Portuguese and indigenous languages [140]. Currently, half of the human population speaks one of some 25 languages of a total of circa 7000 languages; conversely, as little as 0.1% of the world's population currently speaks one of ∼3500 languages [141,142]. There is a double challenge here: (1) how to access this information in other languages, not only if it is published but primarily if it is transmitted orally; and (2) it has been estimated that some 30% of the 7400 languages in this world will be extinct by the end of the 21st century [143]. This language extinction is crucial because three-quarters of the medicinal plant applications are only known in a single language, often threatened with extinction. Hence, they concluded that language disappearance would be more critical to medical knowledge than biodiversity reduction. How can the ongoing extinction of languages and the concurrent loss of ecological knowledge and information unique to those languages be counteracted [144]? In a survey of the fate of local or indigenous knowledge reported in the literature to identify drivers of knowledge evolution across the world, seventy-seven percent of the cases reported a loss of ecological knowledge, followed by 14% of the cases in which a persistence or transformation without loss of knowledge took place [145]. A few studies cited [145] estimated an annual loss rate of ecological knowledge ∼2%. Medicinal and ethnobotanical (other plant information except medicinal) knowledge were the types most impacted. Economic shifts from primary to secondary sectors, rural exodus and urbanization processes, deforestation and modern agricultural practices were deleterious to local ecological knowledge. In contrast, the persistence of knowledge could be related to internal processes such the persistence of belief systems and knowledge transmission patterns, or external processes such as the maintenance, revitalization, or adaptation of traditional practices, some form of integration into the market place, ecotourism, the creation of local cooperatives, and local education [145], in addition to plant conservation-focused measures such as *in situ* conservation and seed banks.

### Biodiversity — diet diversity

One of the persistent questions regarding biodiversity and its relationship with the human condition is whether there is a correlation between biodiversity availability and dietary diversity. The complementary composition of crops originating in the different regions of agricultural origins (for example, between grain legumes and cereals [13]) suggests that the choice of food domesticates was not random but may have reflected agronomic and dietary considerations. Currently, there is an increasing emphasis on diversification of human diets, mostly plant-based, to counteract in part the emission of greenhouse gases, the spread of unhealthy diets based on refined sugars and fats, oils, and meat leading to chronic human diseases, and the narrowing of crop diversity [98,146].

An increasingly diverse agricultural supply may stimulate the diversification of human diets. In India, more than 20 cropping systems are practiced [101]. The two most important ones are rice (*Oryza sativa*) — wheat (*Triticum sativum*) and rice — rice. The rice-wheat system in the Indo-Gangetic plains enhanced food and nutritional security but displaced grain legumes, led to declines in productivity, and increased micro-nutrient deficiency [147], who suggested that cropping system diversification with grain legumes and vegetables would increase dietary diversity and enrich soil health. In Malawi, farm production diversity was associated with dietary diversity, especially in women-headed households. Grain legume, vegetable, and fruit consumption were associated with greater farm diversity [148]. However, the relationship between production and dietary diversity is highly complex and depends on numerous factors. In addition to gender, it depends on wealth, market access, and the specific nature of farm diversification [149]. In 19 of 21 studies, a generally small, positive association was observed between agricultural biodiversity and diet diversity [150]. At very low levels of agricultural biodiversity, an increase in agricultural biodiversity was associated with sharply higher diet diversity, whereas, at moderate and high levels of agricultural biodiversity, the same increase was associated with no change and lower diet diversity, respectively. Hence, it can be concluded that, generally, there is a positive relationship between agricultural biodiversity and dietary diversity, but the magnitude is variable and tends to be small.

Finally, many of these studies consider biodiversity at the species level but ignore intra-specific diversity, which is of interest in crop improvement and plant breeding. A diverse diet, based mainly on plant-based food, may reduce chronic, diet-related conditions and the nutritional quality of cultivars becomes essential [101].

## Trends in crop biodiversity

Various natural and anthropogenic factors affect trends in biodiversity at successive stages, which are discussed from the perspective of crop genetic diversity.

### Long-term evolution before the Holocene

Individual species’ biological and genetic characteristics were largely predetermined during an evolutionary period pre-dating the domestication phase of the transition to agriculture at the end of the Pleistocene, i.e. the end of the last Ice Age [12]. Both genetic processes and environmental factors play a role in shaping the diversity and distribution of crop plant ancestors. The original distribution of the wild progenitor of crop plants evolved before domestication based on dissemination influenced by climate and animal vectors.

Whole-genome duplications (WGD) leading to polyploidy have been a particularly prevalent phenomenon in Angiosperms despite the initial negative consequences of chromosome doubling [151–153]. Yet, ancestral polyploidizations are linked to the origin of major lineages such as the core dicots, monocots, and botanical families (legumes, composites, grasses, and orchids) and associated with striking periods of climate or geologic change (e.g. Cretaceous-Paleogene extinction: [149,154]), which likely involved major stresses. In polyploids (including their diploidized descendants), increased genetic variation due to gene duplications and interactions may have promoted phenotypic diversity of different kinds, including resilience to new biotic or abiotic stresses [153–155]. From a domestication standpoint [156–158], this novel phenotypic diversity generally arising from recent polyploidization events has also played an essential role in extending the adaptation of crops and creating novel traits. Hexaploid wheat (*Triticum aestivum* AABBDD genome) has the unique property, not found in its two parents (*T. durum* AABB genome; *T. tauschii* D genome), of producing gluten, an elastic protein matrix trapping gases produced by yeast [159]. Another example is the tetraploid New World cottons (*Gossypium hirsutum* and *G. barbadense*, AADD), in which most factors improving fiber quality and yield are located on the D genome, derived from an ancestral species that does not produce fibers. So, in the evolution of plants, WGD and polyploidization have played an outsized role in their diversification and, ultimately, domestication [160,161]. Not all plants, however, have been subjected to recent polyploidization; other factors have also played a role in their phenotypic diversification.

Major geological events like glaciations can affect the distribution and genetic diversity of populations. A recent study [162] examined a previous observation that species in tropical areas contain higher levels of intra-specific genetic diversity, mirroring geographic gradients in species richness. Using nucleotide diversity of mitochondrial DNA (animals) and chloroplast DNA (plants), they extended these analyses to 38 000 vertebrate, insect, arachnid, and plant species. They confirmed a latitudinal gradient with higher intra-specific nucleotide diversity in the tropics in vertebrates and plants but not insects. Their data were consistent with the effects of Quaternary glaciations, which reduced population size through genetic drift, mainly in temperate species, followed by population expansion after the end of glaciations. As far as plants are concerned, tropical regions are more species-rich and contain higher levels of genetic diversity because of demographic changes associated with Quaternary glaciations. Four local plant taxa showed high species richness and a higher frequency of endemic species at elevations of 2500–3500 m in the Amotape-Huancabamba Zone (AHZ), situated between 3–8°S. Lat. in the Andes of northern Peru-southern Ecuador [163]. The authors hypothesized that the higher frequency of landslides at these altitudes would result in habitat heterogeneity and, therefore, higher genetic diversity. In contrast, the biodiversity patterns observed did not reflect the orogenic uplift.

The extensive distribution of the common bean ancestor, which extends from northern Mexico to northwestern Argentina over ∼10 000 km, is the result of rare long-distance, bird-mediated dispersal events from Mesoamerica to the Andes dating back to some 400 Ky ago [164]. One of the events led to a *Phaseolus* speciation event (*P. debouckii*) in the AHZ mentioned earlier. The time frame precedes by many years the eventual arrival of humans in the Americas, who would eventually domesticate common bean in Mesoamerica and the southern Andes [44,165–167] Gepts and Bliss [164]; This geographic divergence has had consequences for bean breeding (Andean vs. Mesoamerican divergence: [167–170] and bean pathogen diversity [171,172].

These examples illustrate how the Earth's geological history — especially its more recent one before the Holocene — affects biodiversity and should be considered in conservation efforts, whether *in situ* or in botanical explorations. A further impetus towards conservation is the observation [173] of a decline in within-population genetic diversity in some 91 species of wild organisms since the Industrial Revolution, a further indication that the Earth has entered the Anthropocene era [174].

### The original process of domestication during the transition from hunting-gathering to agricultural production

It is generally accepted that domestication and subsequent evolution processes have led to a marked loss of genetic diversity, especially at the intra-specific level, a phenomenon labeled genetic erosion by the late E. Bennett [175,176]. The latter is, however, not general, and significant exceptions have been documented [177]. Overall, landraces have suffered widespread losses over the last century, with 86% of studies reporting declines of various types, including the disappearance of specific landraces and crop species, reductions in richness, losses of intra-landrace diversity, and declines in the number of farms or villages (see ref. in [177]). Concurrently with these biodiversity losses are the losses in traditional knowledge about these landraces, as mentioned earlier in Functions and uses of biodiversity [34,178].

**Figure 3.**
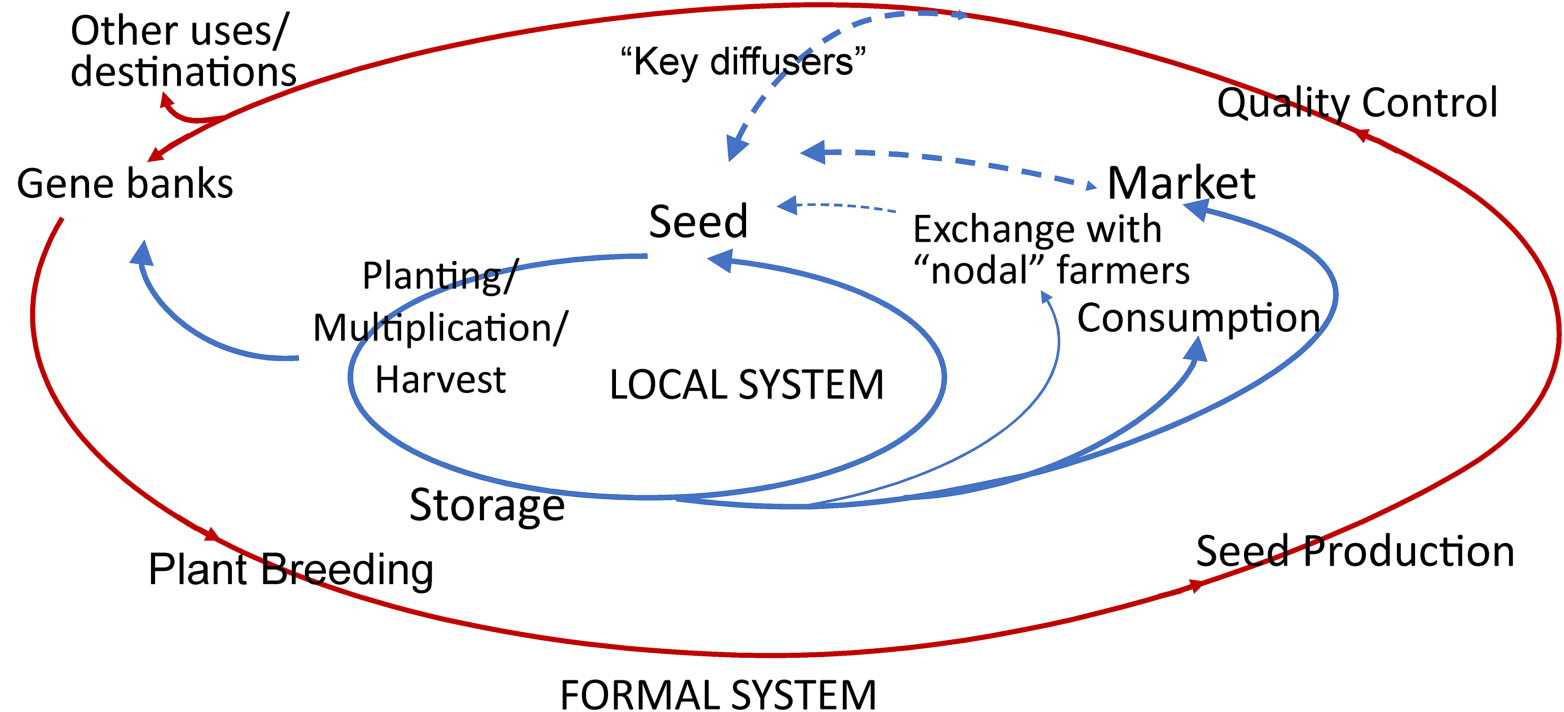
Co-existence of formal and informal seed systems. Multiple potential sources of crop biodiversity are available to farmers, especially in centers of crop diversity and the possibility of gene flow among different crop varieties (e.g. the process of creolization [120,128,183]). Red: formal seed system; blue: informal system. Permission received from [184]. Figure modified from [185].

In examining the drivers of the genetic diversity status of landraces, it is essential to realize that a complex array of factors comes into play. The drivers of this overall reduction are mainly the replacement of landraces with modern cultivars, such as in the U.S. Corn Belt, where a nearly complete replacement took place, especially between 1925 and 1950 [177,179]. Later in the U.S.A., the Southern corn leaf blight, a fungal disease of maize, struck across a major part of the Corn Belt in 1970. The widespread susceptibility of the maize seed stock was due to the uniformity of a mitochondrial DNA gene, which had been introduced to cause genetic cytoplasmic male sterility in a hybrid seed production system. Inadvertently, this mitochondrial factor also caused susceptibility to a toxin produced by the causal fungus (*Bipolaris maydis* (Nisikado & Miyake) Shoemaker) [180]. In Mesoamerica, in contrast, a partial replacement is taking place mainly in lowlands (<∼1400 m) accompanied simultaneously by maintenance or enrichment ([177], Figure 3). Other drivers include agronomic factors, demography (e.g. urban migration), land use or abandonment, environmental changes including human-induced climate warming, and agricultural and economic development. In recent millennia (post-domestication), many crop wild relatives have suffered a reduction in genetic diversity because of habitat loss, gene flow from sympatric domesticated types that are depauperate in diversity (e.g. common bean [181,182]), and unfavorable agronomic practices near fields.

As a counterpoint to this overall reduction trend, there are localized increases in diversity depending on seed exchanges in traditional ecosystems, gene flow with sympatric wild relatives, and adoption of and hybridization with improved cultivars, also called *creolization* or *acriollamient*o (e.g. Mexico; common bean [127,128]; maize:[183]). In regions of domestication, where subsistence agriculture is practiced, landraces persist because of their adaptation, stable and resilient production in the face of stresses in heterogeneous environments, and integration into local cultures (e.g. culinary and dietary preferences) and markets [177]. A caution here is that landrace names are generally unreliable indicators because of synonymy and homonymy and, hence, poor correlation between name and genetic diversity.

Species interactions form an additional dimension of biodiversity. After domestication, pollination and natural pest control have been impoverished [187]. Reversing these trends in biodiversity interactions should be part of reestablishing biodiverse agroecosystems.

### Continued evolution under cultivation post-domestication

#### Hybridization between conspecific wild relatives and domesticates or among domesticates

Because domesticates and their wild ancestors or relatives generally belong to the same primary gene pool [188], hybridization between the two types of populations in sympatry [189–191] or among domesticates [192] can be expected and have been widely documented in numerous crops, especially since the development of genomics and re-sequencing of large number of accessions within crop species.

The consequences of hybridization are varied, ranging from the appearance of new wild taxa and eventually new domesticates of the same or increased ploidy, adaptive introgression, transient hybrid swarms (e.g. [193]), appearance of noxious weedy relatives (e.g. wild maize or teosinte in Europe [194]; weedy rice [195,196]; sorghum [197]), and genetic swamping or extinction by hybridization [198]. Examples of new taxa and crops (see also Long-term evolution before the Holocene) include wheat, cotton [157], *Brassica* sp. [199], strawberry (*Fragaria* spp., [200]), and the year-long bean (*Phaseolus dumosus* [201–203]).

There are numerous cases of adaptive introgression [204]. A well-documented case is the adaptation of maize to highland Mexico after its domestication from *Zea mays* subsp. *parviglumis* in western Mexico at medium altitude, through hybridization with a local teosinte or wild maize, *Z. mays* subsp. *mexicana* [205]. Hybridizations can improve the adaptive potential of predominantly clonally propagated crops like cassava (*Manihot esculenta*) [206]. Common bean can incorporate loci from different taxa, even distant ones, through outcrossing and asymmetric introgression events leading to the acquisition of adaptive traits from wild relatives [170].

Overall, these hybridization events increase allelic diversity if they result in selective introgression, conferring a broadening of the adaptation. They also increase the number of genetic combinations after recombination. Genomics plays an essential role in identifying introgressed regions and candidate genes contained in them that are associated with introgressed traits, including candidate or causal genes, responsible for the traits of interest, which can then be introduced in improved cultivars [204,207,208].

#### Land use changes

The Anthropocene has witnessed tremendous land use change, which is one of the significant causes of biodiversity loss. Nearly a third of the global land area had been affected in barely six decades from 1960–2019 [209]. This is about four times larger than had been previously reported. Land use changes in the global North (including China) represented afforestation and cropland abandonment. In contrast, in the global South, this represented deforestation and agricultural expansion and exportation for beef, sugarcane, and soybean production in the Brazilian Amazon, oil palm in Southeast Asia (see also [210]), and cocoa in West Africa. Cropland expansion affects biodiversity hotspots mainly in Central and South America, while cropland intensification threatens biodiversity in South and East Asia and Sub-Saharan Africa. Thus, agricultural production gains (and the concomitant biodiversity loss) will occur predominantly in developing tropical regions where most of our crop biodiversity originated. A glimmer of hope is offered by an observed deceleration in the latter part of this period (2006–2019) due to the possible effects of global trade on agricultural production and the recent decline of large-scale land acquisitions [209].

Recent demographic and economic changes, such as migration from rural to urban areas and the development of large plantations (e.g. oil palm [210]), have led to changes in land use. These changes sometimes lead to land abandonment, ecological succession, and net forest area gain. Although some may interpret this land use change as a gain in biodiversity, this decline in land use activity may lead to a localized reduction in biodiversity because of a reduction in the forest-agriculture mosaic [211]. The agroecosystems maintained by indigenous cultures, such as the Zapotecs and Chinantecs in Oaxaca, Mexico, should be seen as agents of adaptive biodiversity and cultural landscape maintenance [212].

Although most biodiversity restoration activities have focused on the elements of biodiversity at the population, species, and ecosystem levels, a vital conservation strategy should focus on the human element of biocultural diversity [211]. The maintenance of the highly biodiverse landscape mosaics and their species richness and intra-specific diversity depends on keeping people on the land and limiting rural exodus: ‘If you do not use it, you lose it’ (e.g. Lima bean [135]). This can be achieved more efficiently by providing suitable infrastructure, such as good roads, markets, medical facilities, and schools, to reduce the temptation of rural exodus. A combination with crop diversification can increase farmer welfare and achieve other goals such as anthropogenic global warming (e.g. Kenya [213]).

Another potential cause of biodiversity loss is the weedy nature of some crops and their wild progenitors. This weediness reveals itself through an adaptation to open, disturbed environments, such as agricultural fields and roadsides, as evidenced by the presence, for example, of crop wild relatives within the fields or at their edges. Land abandonment sets in motion an ecological succession, but in many cases, the eventual fate of biodiversity in these abandoned fields is unknown or difficult to predict. Restoration of the original vegetation depends on whether biotic and abiotic thresholds have been crossed during cultivation, which determine if the succession will lead to the re-establishment of the regional species pool with a diversity of life histories, the maintenance of a degraded state, or even favor the establishment of invasive species [214]. Hence, land abandonment does not necessarily return land to a biodiverse state.

#### Socio-cultural organization and seed systems

In the farming systems developed after the transition from hunting-gathering to agriculture, farmers continued to shape the diversity of the crops they cultivated, up to this day, in a traditional context. Farmers’ actions, like introductions and selections, reflect socio-cultural differentiation and economic contingencies; they act upon variability generated by hybridization among landraces, gene flow between crops and their wild relatives, seed exchanges and seed systems, and seed banks (i.e. local crop biodiversity collections). Ultimately, they shape variations that respond to the needs of farmers/consumers for suitable agronomic (e.g. flowering time and climatic and soil adaptation), culinary needs (e.g. cooking time, cooked product quality), dietary needs (e.g. calories and proteins), and affordability to consumers.

The morphological diversity of maize landraces in Cuzalapa, an indigenous community in the Biosphere Reserve of the Sierra de Manantlán in western Mexico, results from the combination of local landraces, varieties from farmers in other communities, and improved cultivars obtained by plant breeders [215]. Selection by local farmers appeared to focus primarily on ear characteristics, leaving other traits to vary following hybridization [216]. The effectiveness of farmer selections can be identified by analyzing farmers’ diverse seed stock, including strongly heritable phenotypic traits, molecular markers like microsatellite markers, and, increasingly, DNA sequencing. For example, the sequences of two of three genes involved in flowering time showed a positive selection among domesticated types but not wild types of pearl millet (*Pennisetum glaucum*) [217]. This genotypically controlled phenological diversity contributes to the stability of production in the Sahel despite variable yearly rainfall.

Correlations between single-nucleotide polymorphisms (SNPs) and climate and soil gradients of the environment of origin in a large set (*n* ∼ 1950) of georeferenced sorghum landraces identified SNPs associated with these environmental variables, independent of geographic distance, and confirmed the associations with drought stress and aluminum toxicity under controlled experimental conditions. The enrichment of environmental associations in genic SNPs suggests that actual genes were — at least in part — involved in environmental adaptation rather than by mere linkage disequilibrium. Although the authors did not document a direct role in farmer selection, farmers will likely select those landraces that show adaptation to local soil and climatic conditions [218].

One of the least studied aspects of landrace biodiversity is the temporal evolution of genetic diversity. A rather extreme case, which deserves further study across a broader range of crops, is the case of Lima bean (*Phaseolus lunatus*) in the Yucatan Peninsula, based on collections made in 1979 and 2007 [219]. Overall levels of genetic diversity, assessed with microsatellite markers, decreased between the two collections; the analysis also showed a qualitative reduction in diversity with a displacement of the original alleles present in 1979, possibly by the introduction into the Yucatan Peninsula of improved varieties or landraces. Phenotypically, the accessions of 2007 also showed a markedly larger frequency of white seeds, further evidence of the turnover revealed by the DNA marker analysis. Along similar lines, the introduction of the single clone ‘azul’ (‘blue agave’) of *Agave tequilana*, adopted for the marketing of tequila, has caused losses of landraces involved in the production of agave spirits in west-central Mexico [220].

The latter citation points to exterior forces affecting farm, regional, or national biodiversity. More will be said about this in Legal, policy, and valuation framework. However, a phenomenon that directly affects farm-level biodiversity is the existence of seed systems [118]. Provisioning of seeds is a critical element in human food security and nutrition, but also in agrobiodiversity and genetic conservation. Seed systems transfer seed and other multiplication materials from farmer to farmer by purchasing, gifting, or bartering via the non-commercial and commercial sectors, with substantial porosity between the two sectors (Figure 3). 2009 First, farmer-based seed systems provide a substantial proportion of the seed farmers need. These seeds represent genetically diverse materials within and across crops, as they even include varieties or crops not supplied by the formal sector [117,118,221]. Farmer seed producers can maintain morphological and yield characteristics over multiple seasons (see also, for example, the ‘Seed Engufu’ variety with red mottled seeds and a single DNA fingerprint [120]).

Second, the farmer seed systems are flexible in readily incorporating new crops or domesticates, varieties, and cultivars from the formal seed sector. Seed transfers are combined with transmission of knowledge about the crop varieties, like agronomic management, yield and consumption qualities, and sensitivity to diseases and herbivores. This flexibility assures the long-term survival of informal seed systems and plays a vital role in farmer-based agrobiodiversity conservation [118,221]. Following the construction of a highway in the Palcazu Valley in the upper Peruvian Amazon, the Yanesha people adapted changes in agricultural conditions caused by the new road, which facilitated the flow of plants, people, and markets [222].

Third, some institutions and social relations favor or limit the transfer of seeds or planting materials and the associated knowledge [223]. Crop varieties are social objects and, thus, are embedded in pre-existing social structures and reflect farmers’ social identity [224]. Hence, the behavior and performance of landraces can be considered as the result of a triple set of interactions: G × E × S, where G, E, and S are genetic, environmental, and social differentiation factors, respectively. Examples of S factors are ethnolinguistic boundaries (e.g. maize of the Tzeltals and Tzotzils in Chiapas, Mexico [225]; pearl millet on the western side of Lake Tchad [226], and social relationships, such as neighborhood-groups, clans, age-sets, and marriage (e.g. sorghum of the Tharaka near Mount Kenya [223,227]; *Dolichos lablab* in Colombia, P. Gepts, personal observation).

#### Gender effects

Gendered differences in biodiversity knowledge result from men and women having different tasks in the household and sometimes farming other crops or plots. In addition, women are increasingly important in farming operations because socio-economic changes drive rural exodus by men. Despite these well-established facts, gender differences in the handling, maintenance, and knowledge of biodiversity have been neglected.

Based on their essential role in cultivating maize landraces in home gardens in the Bajío region of Mexico, women have a distinct knowledge of crop biodiversity [228]. Women were growing varieties of maize for household culinary and dietary uses, whereas men were growing other varieties, primarily for producing maize for sale. Hence, maize ears were selected for different qualities. The most important trait was the size of the ear, but other traits were also considered, including color, number, and straightness of rows, lack of insect damage, and ease of removing grains from the cob. Because men and women grew corn varieties separately and for different purposes, the knowledge of both should be considered when conserving this biodiversity.

The Tharaka of Kenya are agropastoralists who rely on crops that tolerate hot and dry conditions and nutrient-poor soils. A mix of methods combining interviews, focus groups, and a household survey [229] led to the observation that the distribution of pearl millet and cowpea (*Vigna unguiculata*) indigenous varieties had been shaped by the migration of the first Tharaka people, some 300 years ago into the South Tharaka region. Further spread of these original pearl millet varieties (but not cowpea varieties) towards the North Tharaka region was limited because the first migrants were male. At the same time, women were the primary keepers of these varieties and the associated knowledge, creating a social barrier to the dispersal of the crop and a differential distribution of pearl millet landraces across the local landscape.

In southern Ethiopia, both women and men ranked earliness as the most desirable varietal trait in common bean varieties grown for their dry grains [230], reflecting a common need of subsistence farmers for whom the last day before harvest represents maximum food insecurity and harvest day maximum food security until the next harvest. Other traits that were ranked similarly by the two genders were drought tolerance and grain yield. In contrast, marketability and germination were particularly valued by men, whereas culinary quality (fast-to-cook and taste) was more valued by women [230].

Thus, gender-based information should be considered when assessing, evaluating, and conserving crop biodiversity because they will likely provide different results.

### The effect of dissemination from regions of domestication into other cultivation areas

Part of the diversification process observed for our crop species results from a complex dissemination process from the regions of domestication to other regions around the world. This process has been so pervasive that the highest production levels are found outside their respective centers of domestication for many crops. Over the past 50 years, crop production and consumption has been based for ∼70% on non-indigenous crops [146,231]. These data indicate that many food crops have been disseminated to other regions, where they were most likely confronted with different environmental (biotic and abiotic) conditions [232,233].

One of the most frequently recurring adaptive traits is day-length sensitivity (photoperiod sensitivity). As many crops originate in tropical or semi-tropical areas [12–14,234], where short-days/long-nights are prevalent, their development and reproduction are interfered with when they are introduced into regions at higher latitudes with long-days/short-nights during the growing season. The interference, often combined with temperature interactions, affects flowering (e.g. common bean [192]) and tuberization (potato, *Solanum tuberosum* [235]). During these episodes of crop dissemination, humans selected mutants that would overcome these environmental constraints; these mutants pre-existed the dispersal (part of the standing variation of the crop or its wild progenitor) or appeared *de novo* during or after dispersal.

An example of the former is the photoperiod insensitivity that characterizes common bean varieties in temperate regions, such as Europe. Through a combination of genome re-sequencing, metabolomics, classical phenotyping, and data analysis for environmental association in a sample of some 220 entries including Andean and Mesoamerican domesticates from the Americas and Europe, several genome regions involved in photoperiod sensitivity were identified [192] among common bean domesticates introduced early on after 1492 from the Andean region into Europe, likely derived from the Andean ecogeographic races Nueva Granada and Chile [236]. This photoperiod insensitivity was further introgressed extensively into Mesoamerican domesticates of the Durango ecogeographic race introduced into Europe, allowing for the subsequent dissemination of these domesticates in the entire continent. One of the Andean genome regions promoting early flowering was a segment on chromosome Pv01, containing the gene for determinacy *PvTFL1y* (*fin*, Phvul.001G189200), confirming earlier results for Europe [237] and China [238], and the linked photoperiod sensitivity gene *Ppd* (*E3/PHYA3*, Phvul.001G221100) [239]

An example of a *de novo* mutation conferring adaptation after dissemination is the gain-of-function adaptive alleles in the *StCDF1* gene conferring tuberization under long days in the European environments [233]. Like the introduction of the common bean in Europe, admixture may have facilitated the rapid spread of adapted potato cultivars, resulting in broader domesticated gene pools.

Other factors play a role in adaptive selection determining the geographic dissemination of rice (*Oryza sativa*) in East and South Asia [240]. These include migration barriers (such as mountains or seas) or geographic distance, abiotic variables (temperature, moisture, and soil types), linguistic groups, and culinary properties (stickiness). A combination of archaeological records, population genomics, environmental niche modeling, empirical field and laboratory investigation, and ancient Chinese text analyses illustrated the importance of various climate variables in determining the human-mediated cultivation range expansion of mung bean or green gram (*Vigna radiata*) after its domestication in South Asia [241]. In a first step, mung bean was disseminated eastwards to Southeast Asia, then northwards to China, and finally westwards to Central Asia. The authors attribute the late arrival of mung bean in Central Asia to an initial lack of drought adaptation despite the possibility of early human contact between South and Central Asia. Elucidating the dispersal pattern of a crop like mung bean also contributes to identifying germplasm with valuable traits like potential drought tolerance. Indeed, the Central Asian mung bean accessions exhibited a higher root:shoot ratio, a phenotype associated with lower water availability in grain legumes (e.g. [242]).

Because of the importance of the evolutionary and historical components, the research into domestication and subsequent dissemination involves — whenever available — ancient DNA from different sources, including archaeobotanical remains and herbarium specimens. Combining sequencing of fifteen ∼2000-year-old maize cobs from southeastern Utah on a temperate plateau and genomic predictions established on a modern maize panel, archaeological maize varieties did not appear to be well adapted to their local environments: they were late-maturing (subject to early frost), short-statured, tillering, and segregating for yellow kernel color (the latter a derived, nutritionally important trait, which may have evolved *de novo* [243].

In summary, mutations, whether *de novo* or part of the standing variation, and hybridizations led to novel gene combinations, generally distinct from those observed in the respective centers of domestication. These combinations, selected by humans, allowed the adaptive, human-mediated spread and establishment of crops in their novel environments where they eventually took on important social, economic, and cultural significance (e.g. Europe: [244–248]). In turn, associations between these novel genotypes and phenotypes can lead to identifying valuable germplasm for crop improvement.

### Contemporary cropping systems and breeding since the early 20th century

Agricultural land management systems reflect a wide range of cropping systems and the various intensities in which crops are grown ([113]: their Table 1). It is generally held concern that modern cropping systems, in general, and plant breeding, in particular, have reduced the genetic diversity of standing crops because of selection and replacement of traditional landraces by modern cultivars. Several studies have examined this tenet and have come to mixed conclusions. Contradictory forces affect crop genetic diversity [249]. On the one hand, an economics of scale operating that lowers the unit cost of production due to crop uniformity (e.g. shared phenology, plant, seed, or fruit size, shape, and composition). On the other hand, other economically valuable traits promote the use of more diverse crop cultivars to confer resistance to biotic and abiotic stresses, other crop production traits, and technological or consumer demands that vary over time and space.

**Table 1:** Summary of organic agroecological pest management practices (modified from Brzozowski and Mazourek 2023; sources therein)

	Practice or trait	Results
Plant-based resistance	Physical traits	Deter or impede mobility of insect pests or colonization of plant pathogens (i.e. cuticle composition
		Canopy architecture can shade weeds, or alter environmental conditions (i.e. humidity) to slow pathogen growth
	Chemical traits	Volatile deterrents for insect pests
		Harmful or deterrent secondary metabolites for pathogen and insect pests, and allelopathic compounds inhibit weed growth
		Volatile cues for insect predators or parasitoids about location of prey
		Qualitative gene-for-gene interactions or quantitative traits
	Genetic tolerance	Plants exhibit no apparent yield or fitness cost to pest damage
Farm-scale cultural practices	Sanitation	Clean planting material and equipment stop inoculum from entering farm (pathogens, weeds and insects)
	Crop rotation	Disrupt pest lifecycles (pathogens, weeds and insects)
	Applying botanical diversity	Trap crops or push-pull systems rely on differential plant attractiveness to lure and, or repel insect pests from main marketable crop
		Provide habitat and alternate food sources for plant beneficial insects
		Modify epidemiological factors to slow the spread of pathogens through crop rotations, intercropping, companion planting or growing a crop mixture
Crop targeted interventions	Beneficial organisms	Beneficial insects that are predatory on pests, and nematodes and effective microbes can further suppress insect pest and pathogen populations
	Mechanical interventions	Cultivation, thermal and mechanical measures to manage weeds or pathogens
		Specific passive traps (like trenches) or active control like vacuuming to manage particular insect pests
	Naturally derived products	Non-synthetically derived products like oils, soaps, or extracts, can be used to supplement pest management efforts

In a study of DNA marker diversity of existing Canadian flax, oat, wheat, potato, and canola gene pools 100 years before 2009, a significant allelic reduction starting in the 1930s in the wheat gene pool was observed [250]. This situation arose because breeding was limited to three significant introgressions over time, and the selection for bread wheat quality traits intensified. An allelic loss of diversity was also observed in the oat gene pool starting after 1970. Genetic erosion was observed in the flax gene pool over 90 years, especially after 1947 because of the intensive selection for rust resistance. These same authors observed a narrow genetic base in potato despite using several potato germplasm groups (*Phureja* and *Andigena*, in addition to *Tuberosum* and several wild potato species). In canola, a low-erucic acid, low glucosinolate version of rapeseed, there has been an overall reduction in allelic diversity over the 60 years of breeding rapeseed. However, the introduction of canola varieties did not affect overall genetic diversity. The authors conclude that generally, there was a pattern of reduced diversity in Canadian breeding efforts in this mix of cereal, oil, and specialty crops but with differences in magnitude, pace, and timing.

These results contrast with those about the spatial and temporal diversity of the U.S. wheat crop using phylogenetically unaware vs. informed measures of biodiversity [251]. The former does not consider the genetic relationships among varieties, while the latter does. Using phylogenetically unaware measures, the authors observed increased spatial and temporal diversity, including an acceleration of varietal turnover over the past century. Phylogenetically informed measures detected an increasing variation over time, especially in winter wheat, compared with durum or spring wheat. The phylogenetic background of wheat varieties also changed over time following variety turnover. The analyses also identified geographic groups of cultivars, presumably reflecting differential adaptations. These were Central Plains, Northern Plains, Southeast, and the Pacific Northwest for winter wheat. For spring wheat and durum wheat, the Northern Plains, and the Pacific Northwest. Thus, overall, the two types of diversity indices increased over time for all three market classes of wheat in the U.S. The authors concluded that the increasing use of bread wheat varieties, developed mainly by the public sector, led to more biodiverse cropping practices.

The Green Revolution research in cereals (wheat, rice, maize) and other crops (cassava, lentil, beans, and potatoes) over the period of 1961 to 2004 saved ∼18–27 million hectares from being brought into cultivation [252]. Most of the saved land would have been in developing countries, displacing tropical pastures and forests and representing an additional threat to biodiversity. However, whether this increased production would have saved land or encouraged agricultural area expansion deserves further study. Agricultural expansion occurred in Africa (59%, from 32 to 51 million hectares) and Asia (34%, from 177 to 237 million hectares); the total expansion was primarily associated with the planting of improved cultivars at the expense of landraces. Concurrently, the area planted to landraces decreased markedly (88%) in Asia, from 156 to 19 million hectares; in contrast, landrace area decreased only slightly (9%) from 32 to 29 million hectares in Africa [253]. Participatory breeding led to the development of three modern rice varieties in highland Nepal, leading to the first introduction in 1996 [254]. Just eight years later, up to 60% of the land area was occupied by the three modern varieties. Although seven landraces had been abandoned during the modern variety introduction, other diverse landraces were still grown on up to 40% of the field area. The maintenance of these landraces answered to several needs of farmers and consumers not provided by the modern varieties (see Socio-cultural organization and seed systems).

Finally, although there is a need for crop diversification, some obstacles exist in this process. In the U.S., at the county level, the factors most predictive of crop diversity are climate, land-use norms, and farm inputs [255]. There are, nevertheless, regional differences in the relative importance of these factors. Thus, crop diversification requires consideration of factors other than mere agronomic adaptation, high performance, and farmer and consumer acceptance.

In conclusion, on trends in crop biodiversity, there is incontrovertible evidence the biodiversity in agricultural ecosystems has been declining [17]; a diagnostic of the causes of this decline can lead us to the development of a variety of solutions at the field, landscape, regional, and global levels.

## The use of biodiversity in cropping improvement

**Figure 4.**
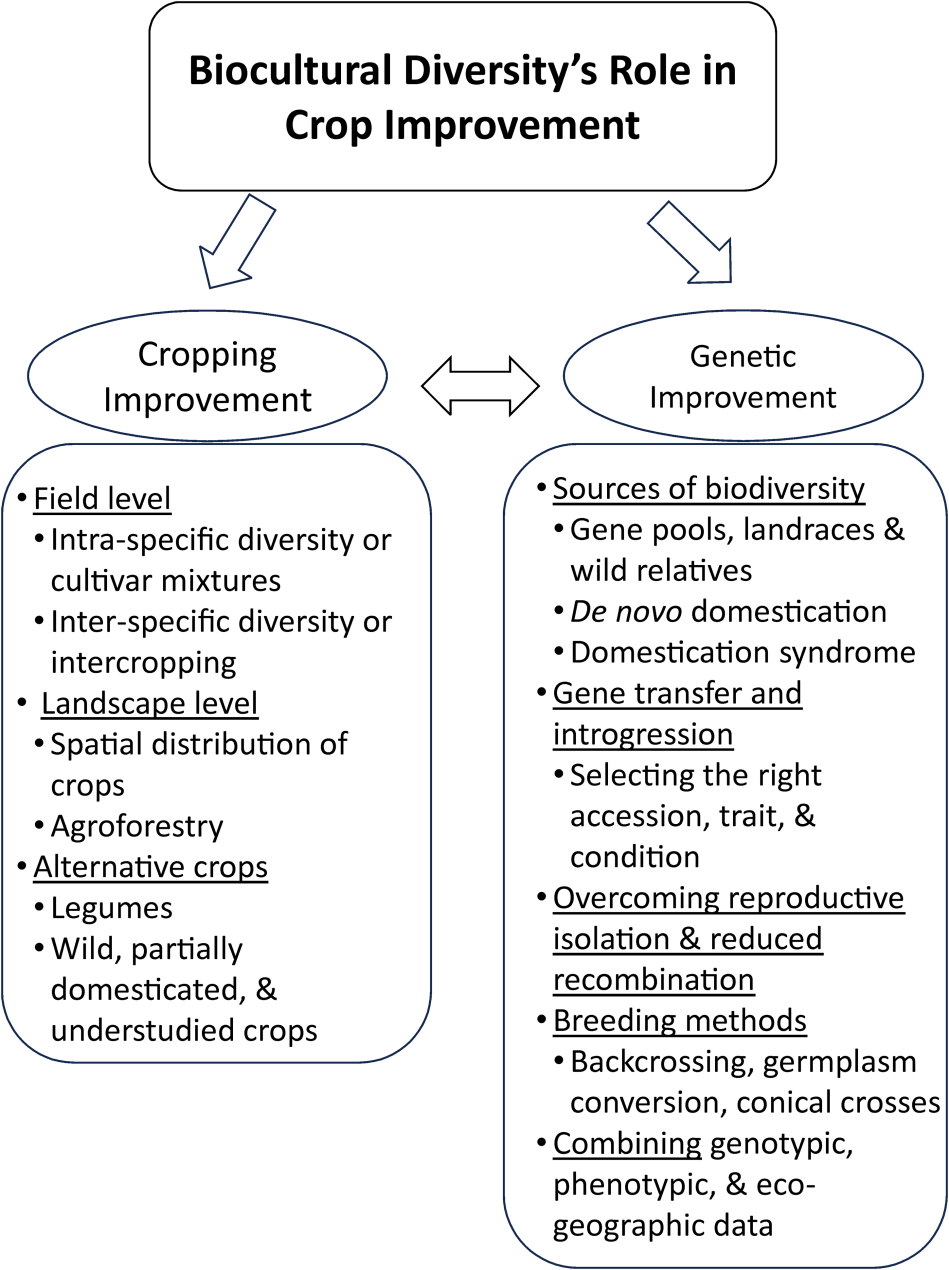
The two main ways in which biocultural diversity is used in crop improvement. These include cropping improvement (also known as agronomic management, see The use of biodiversity in cropping improvement) and genetic improvement (a.k.a. plant breeding, see The use of biodiversity in genetic improvement). The figure shows the different topics discussed in the two sections. The challenge for agronomists and breeders is to jointly design management (M) and genetic (G) approaches for non-stationary environments (E), given that rapid climate change, including global warming, presents an added challenge, hence, G × M × E.

In crop improvement, two broad categories of actions can lead to improved performance, including higher yield *per se*, increased tolerance of biotic and abiotic stresses, improved quality of the harvested product, enhanced resource use, and reduction in the environmental impact of cultivation. These categories are cropping improvement (or improved cultivation practices) and genetic improvement (a.k.a. plant breeding) (Figure 4) [256]. Cropping improvement seeks to improve production through how crops are managed; it involves practices affecting planting (e.g. date, density), fertilization (before and during crop growth), disease, pest, weed control, irrigation, and harvest. Genetic improvement seeks to improve production by developing genotypes or populations with productivity or quality potential under specific crop management and environmental conditions. There is often a strong interaction factor between cropping and genetic improvement, both subject to environmental conditions [257]. The challenge for agronomists and breeders is to jointly design genetic (G) and management (M) approaches for non-stationary environments (E), given that rapid climate change, including global warming, presents an added challenge, hence, G × M × E. This section offers several aspects of cropping improvement that integrate crop biodiversity into cultivation practices (M). The use of biodiversity in genetic improvement will focus on plant breeding practices (G) that integrate additional biodiversity.

### At the field level: spatial distribution of genotypes

Intensive agricultural systems generally involve simplified agroecosystems based on monocultures with high inputs of petrochemical fertilizers, pesticides, and herbicides, with attendant adverse effects that include polluted and restricted water supplies, impaired air quality, soil degradation, and biodiversity loss. Increasing crop biodiversity in agroecosystems has been proposed to address these industrial monoculture issues. There are several ways in which crop biodiversity can be increased.

#### Intra-specific diversity or cultivar mixtures

Increasing diversity by introducing intra-specific diversity via multi-lines, cultivar mixtures, or population cultivars has been proposed to achieve higher yield, sustainability, and resilience. The idea of explicitly developing this type of varieties by evolutionary breeding approaches has a distinguished track record [258–260].

A French farmer-led initiative grew a mixture of four bread wheat landraces (called ‘Mélange de Touselles,’ MT) distributed across a farmers’ network of 15 locations, representing diverse environments (e.g. altitude, rainfall) and farming practices (field size, phenology). Space-time samples were also genotyped with 17 microsatellite markers and planted in a common-garden experiment. Results showed a rapid differentiation among populations in different locations, which was larger at the phenotypic and putatively selected (flowering time markers) level than at the neutral genetic level, suggesting that MT contained sufficient standing genetic diversity to allow divergent evolution [261]. Subsequent progress in participatory breeding projects involving farmers, facilitators, and researchers showed that these population varieties’ grain yield and protein content were more stable over space and time than commercial varieties. Protein content — an essential bread-making characteristic — was more stable and correlated with within-variety genetic diversity. Thus, these wheat varietal mixtures were more stable over time, a sought-after characteristic for farmers [262].

In a meta-analysis of 91 studies, yield increased by 2.2% overall in cultivar mixtures compared with the individual components of the mix. Larger mixtures and those with more functional trait diversity showed higher relative yields. Biotic stresses (e.g. diseases) and abiotic stresses (e.g. low soil organic matter and nutrient availability) also increased relative yields. Cultivar mixtures also increased yield stability compared with genetic monocultures, for example, in response to year-to-year weather variation [263].

One obstacle to the more widespread adoption of this multi-line approach may be the legal requirement for uniform lines (DUS: Distinct, Uniform, and Stable) required by the intellectual property legislation (see Legal, policy, and valuation framework). Another obstacle is a logistic one associated with the planting and harvesting on a large scale of polycultures. Hence the interest in diversification strategies relying on within-field crop varietal mixtures. In a review of monocultures, polycultures, and varietal mixtures for six parameters (yield, pest control, ease of implementation, profitability, labor savings, and human nutrition). Polycultures in general enhanced many services, but production costs tended to be high. Monocultures had the lowest production costs but were poor producers of some services, such as human nutrition [264]. Varietal mixtures could address some limitations of mono- or polycultures, but some knowledge gaps remain. These include an understanding of the suitability of varietal combinations depending on the type of crop (other than cereals), the kind of constraint (e.g. biotic: herbivores and beneficial insects), the number and type of varietal components, the target agricultural systems (from subsistence to small-scale to large-scale commercial). In addition, the functional and ecological mechanisms of diversification need to be further investigated, as do the profitability and human nutritional consequences of this type of intra-specific diversification [264].

#### Inter-specific diversity or intercropping

In general, species interactions govern biological communities and their diversity. Some interactions have negative interactions (competition), whereas others are positive (e.g. mutualism or symbiosis). Both abiotic and biotic stresses affect species interactions [265]. Cropping systems based on species mixtures may have several potential advantages in temperate and tropical conditions. These multi-species systems — from soil micro-organisms to plant cultivars and cropping systems — may include annual and perennial species, from two to multiple crop species [266]. A literature survey noted several potential advantages, including higher overall productivity, better control of pests and disease, enhanced ecosystem services, and greater economic profitability (as indicated for natural ecosystems as well, Functions and uses of biodiversity), based on a complex set of spatial and temporal interactions of the above-ground (canopies) and below-ground (soil) compartments [267].

Plant species diversity promotes the productivity, stability, and resilience to disturbance of natural ecosystems. A comparison of the biomass productivity of seven perennial forage species planted in monoculture or two to six species mixtures over three years and under three management intensities (one to three harvests per year) showed a complementarity among species — possibly between legumes and grasses — led to increased biomass yield under all managements and consistently across years [81]. Multi-species combinations were more productive than monocultures when exposed to drought, regardless of the number of — varieties present. In contrast, the temporal stability of production increased only with the number — of varieties present, whether under drought or well-watered conditions, but was not affected by the number of species present. The authors concluded that both types of diversity (species richness and intra-specific diversity) should be included in plant breeding programs destined to boost the productivity and resilience of managed pastures [268] (see more in The use of biodiversity in genetic improvement).

The positive effects of diversity could be due to functional complementarity among species based on complementary resource use. One of the best-known agroecosystems is the milpa system [269–273], consisting of beans (*Phaseolus* sp., maize, squash (*Cucurbita* sp.), and quelites (edible greens). This cropping system may have originated before the transition from hunting-gathering to agriculture, as Mesoamerica's wild ancestors of the three major constituent species overlap. It has sustained pre-Columbian societies in the Americas for millennia because of its dietary and agronomic complementarity (e.g. [274]). It is now distributed not only in Latin America but also in Eastern Africa. The main crops have complementary properties, including root system architecture, nitrogen acquisition and use, and grain nutrient content, which leads to higher overall yields than the crops individually. Differences in root architecture among the three crops lead to spatial niche complementarity, which allows this polyculture to yield above monocultures when mobile resources like nitrogen are limiting [275,276]. Further research is needed to determine to what extent genotypic diversity within the three species can maximize the productivity and benefits of this agroecosystem. Traits that increase the complementary interactions between species should be emphasized, for example, crop cycle length (short-season vs. long-season). Furthermore, following an initial selection of genotypes under monoculture, subsequent selections under intercropping under farmer management should be introduced in the earliest generation possible to identify genotype × cropping system interaction and take into account farmer selection criteria [277].

A meta-analysis of over 45 studies encompassing 500 experiments tested whether crop diversity reduced herbivores and increased natural enemies of herbivores [88]. They found support for herbivore suppression, enemy enhancement, and crop damage reduction using intercropping plantings, the inclusion of flowering plants, and plantings that repel herbivores or attract them away from the crop (trap crops). However, there was a small but significant adverse effect on crop yield, partly because of the reduced area devoted to the crop.

Nevertheless, at the farm or plot levels, biodiversity may not be maintained for purposes other than direct, practical uses and at levels too low to assure many ecosystem services [278]. Interventions at the landscape scale may offer greater improvement opportunities than at the plot scale by increasing integration and resilience across a patchwork landscape of different agricultural uses.

### At the landscape level: spatial distribution of crops

Agricultural landscape homogenization has detrimental effects not only on biodiversity but also on crucial ecosystem services. Various strategies have been proposed to increase crop diversity in space and time to counteract this homogenization while addressing potential trade-offs between biodiversity benefits and agricultural productivity [279].

Can increasing crop heterogeneity across landscapes positively affect overall biodiversity [280]? To this end, they conducted experiments in 435 landscapes belonging to eight contrasting regions of Europe and North America. In each landscape, they sampled plants, insects (bees, butterflies, hoverflies, carabids), spiders, and birds in three sampling sites. Landscapes with increased crop heterogeneity had higher diversity. Decreasing mean field size increased biodiversity even without seminatural vegetation between fields. Increasing the number of crop types increased landscape-level diversity. Overall, their analysis showed that biodiversity in agricultural landscapes can be improved without taking land out of production.

One of the foremost examples of biodiversity at the landscape level is agroforestry, which is especially prevalent in the tropics and is used by more than 1.2 billion people. Agroforestry can address low yields and income by combining shade trees, nitrogen-fixing trees, and indigenous or cash-crop trees that produce nutritious and marketable products, such as coffee and cacao. A high diversity of agroforestry systems still exist worldwide; they are greatly influenced by the socio-ecological context. Generally, agroforestry species combinations restore soil fertility, agroecosystem services, and a source of income while also providing wildlife habitat and reducing the herbivore and pathogen populations. The design of agroforestry system can be integrated into participatory rural development programs, which rely on traditional knowledge but also provide community education and training programs [82,281]. Furthermore, recruiting additional tree species into agroforestry ecosystems via *de novo* domestication would add biodiversity, which would involve clonal propagation for speed and efficiency and a participatory, farmer-driven approach to increase the chances of success [83].

Agroforestry systems can help sustain part of the local biodiversity. In the agroforestry systems of the Tehuacán-Cuicatlán Valley in Central Mexico, 79 species of trees and shrubs have been recorded, 86% being native and representing 43% of the species in the surrounding native vegetation [274]. Trees left standing are used for various reasons, including fruit production, firewood, shade, aesthetics, and respect for nature. Current AFS results from traditional ecological knowledge based on centuries and even millennia of interactions between humans and nature and knowledge and techniques resulting from such interactions. People know the valuable properties of local plants and details about their distribution, year-to-year abundance, phenology, interactions with other plants and animals, and actual uses [282].

One of the central issues is whether there is a relationship between biodiversity levels and yield. In a study of agroforestry systems of 43 smallholder cacao growers in Sulawesi, Indonesia, species richness of trees, fungi, invertebrates, and vertebrates did not show a strong relationship with yield. However, yield depended on the percentage cover by shade trees. Cocoa yield and pesticide expenses did not correlate with the main pest or disease incidence [283]. The authors concluded that specific management practices like weed control and shade trees could achieve joint objectives of biodiversity maintenance and yield increases.

In the tropics, agroecosystems face agricultural intensification towards increasing simplification and expansion at the expense of biodiversity. Cassava is the largest calorie producer among roots and tubers and is grown mainly by resource-poor farmers on marginal lands. A review of 95 cassava intercropping studies across geographies and environmental conditions [284] showed largely positive effects on pest suppression, disease control, and soil and water-related services. Adding maize resulted in 25 positive impacts vs. three negative impacts; adding any of four species of grain legumes resulted in 23 vs. three impacts, and nine vs. 0 for trees. The authors suggested that appropriately designed intercropping systems can balance farm-level productivity, resilience, and environmental health but require transdisciplinary approaches.

Specific features of landscapes also influence biodiversity levels. For example, some landscapes have characteristic dry-stone walls (without cement or concrete) separating fields. These wall types in the region of the northern Apennines (Italy) harbored more biodiversity (measured by lichen, a salamander species, and mollusks), suggesting that this type of wall should be maintained as part of landscape management [285]. Such dry-stone walls are also important to maintain wild crop relative diversity as they provide a protective habitat for seed conservation and plant growth (P. Gepts, pers. observation, Oaxaca, Mexico). Another example is ‘trap crops,’ which attract pests and protect adjacent, less attractive host plants. A mix of three related trap crops (*Brassica juncea*, *Brassica napus*, and *Brassica rapa* subsp. *pekinensis*) had a more substantial protective effect as measured by the yield on the main crop broccoli (*Brassica oleracea* var. *italica*) than each trap crop individually [286]. So, increasing the biodiversity in the field to include not only trap crops in addition to the main economic crop but also increasing the diversity of trap crops should be considered to enhance the approach's success.

Landscapes are rarely managed specifically to suppress pests.A 13-year government data set concerning the European grapevine moth (*Lobesia botrana*) outbreaks and insecticide applications across ∼400 Spanish vineyards showed that, at harvest, simplified vineyard-dominated landscapes showed a four-fold increase in outbreaks compared with complex landscapes in which vineyards are surrounded by shrub vegetation [287]. These results suggest that landscape diversification could reduce pest populations and insecticide applications; wall-to-wall planting of a single crop, like in grape-growing regions or blue agave-growing regions (for tequila production in Mexico), should be avoided to achieve more sustainable productions.

### Focus on alternative, understudied, and new crops or cropping systems

#### The underappreciated legumes: grains, forages, and trees and shrubs

An example of functional utilization of specific crops is the introduction of legumes (Fabaceae), which are known for their positive agronomic impacts, in addition to their significant nutritional impact, especially in third-world countries [288–290] even though they have been neglected over the last decades to the detriment of human health, nutritional security, and sustainable food production [291]. Three main groups of grain legumes are (1) the Inverted Repeat Loss Clade or cool-season clade, which includes pea (*Pisum sativum*), chickpea (*Cicer arietinum*), and lentil *(Lens culinaris*), for example; (2) the Phaseoloid clade (warm season clade), which includes the five domesticated *Phaseolus* beans [44], soybean (*Glycine max*), pigeon pea (*Cajanus cajan*); and the Genistoid and Dalbergioid clades, which include lupin (*Lupin* spp.) and groundnut (*Arachis hypogea*), respectively [292,293]. These different grain legumes have a broad range of climate and soil adaptations, life histories, and reproductive systems and can, therefore, be integrated into wide-ranging agroecosystems. The agronomic benefits are biological nitrogen fixation, weed suppression, erosion control as a cover crop, and soil health improvement, particularly in rotations. The broad diversity of the legume family, particularly its papilionoid subfamily, provides various uses that benefit humans.

An example of the potential of this added emphasis on legumes is provided by a study [294], which compared spatial and temporal crop diversity patterns, including monoculture maize and maize diversified with annual (soybean and ground nut) and semi-perennial, shrubby legumes [pigeon pea and velvet bean (*Mucuna pruriens*)] in temporal and spatial combinations, including rotations and annual or semi-perennial intercrops. Annual cropping was associated with four months of growth, whereas semi-perennials provided an additional two to six months of soil cover. Moderate fertilizer additions doubled grain yield compared with monoculture maize. Across experiments, semi-perennial rotations provided a two-fold superior return, whereas annual rotations yield more modest returns. The authors concluded that there is an urgent need to test legumes that can give specific traits supporting sustainable, productive cropping.

#### Wild and partially or understudied domesticated plants

On a global basis, agriculture is increasingly focused on a limited number of crops, which supply the majority of calories and, to a lesser extent, proteins and other nutrients. It has been estimated that, on average, roughly 2/3 of national food supplies and production are derived from foreign crops, i.e. crops that originated in centers of agricultural origins other than their own. This trend has only increased in the most recent half-century, even in countries with high native crop diversity [98,146]. Broadening our food sources by re-emphasizing partially domesticated (also called understudied plants or ‘Cinderella’ species [83]) and developing new crops (by *de novo* domestication) is increasingly proposed as a wise strategy to assure food security, farm revenue, and sustainable production.

The development of the milpa agroecosystem in Mexico entailed the selection of three primary crops: maize, bean, and squash (see above, Inter-specific diversity or intercropping). Historical sources show that other plants were being domesticated, such as edible greens (so-called quelites, derived from the Náhuatl language) before the start of the European conquest in 1492. For about five centuries, the diversity of domesticated species has decreased by 55–90% despite or because of the cultural importance of quelites. The imposition of European values and the reduction in maize area have reduced their importance, especially in urban areas. Yet, quelites provide critical plant sources, rich in vitamins, minerals, dietary fibers, fatty acids, and antioxidants [295,296]. It should also be remembered that descriptions of native or traditional uses of plants are often written in local languages and not English (e.g. Brazil, Portuguese [140]; Colombia: Spanish [297]. To access information about these alternative wild plants or crops and the associated knowledge, this situation needs to be considered through local collaborative activities.

Bambara groundnut (*Vigna subterranea*) is a close relative of cowpea (*V. unguiculata*). Like cowpea, it originated in Africa but has achieved a much more limited distribution, mainly in that continent, despite its desirable qualities, including yield potential, nutritional composition, drought tolerance, adaptation to marginal soils, and symbiotic nitrogen fixation. The primary gene pool consists mainly of wild types and landraces; only limited breeding for improved cultivars has occurred [298,299]. Like other understudied species, the efficiency of breeding programs would benefit from additional research in the genetic diversity of its gene pools (primary to tertiary), the genetics of its beneficial traits, and a breeder's genomic toolbox.

An example of this approach is the Lima bean (*Phaseolus lunatus*). Despite being the second most important of the five *Phaseolus* domesticates after the common bean, there were very few genetic studies on this species and limited or no genetic and genomic tools. The species is exciting as a potential alternative grain legume under climate change in hotter, humid or dry regions. An international collaboration headed by Colombian researchers led to the first reference genome for this species ([300]: Phytozome id 563: https://phytozome-next.jgi.doe.gov/info/Plunatus_V1; [301]). This achievement included a chromosome-level, high-quality assembly of the genome, the development of a large recombinant inbred population and attendant QTL analysis of agronomic traits, a study of the population structure in the species, and an analysis of gene expression during pod development. An international Lima bean is being constituted, which will provide a multidisciplinary approach towards the improvement of this understudied crop [302]. Similar pursuits are occurring in the other domesticated *Phaseolus* species and in cowpea; therefore, comparing and taking advantage of synteny among these related genera and species becomes possible. Because DNA sequencing is becoming ever more efficient and cheaper, genomic tools are increasingly becoming accessible to understudied plants relatively quickly (see The use of biodiversity in genetic improvement).

Investments in industrial monoculture agriculture in the public and private sectors have established its current yield superiority. The highly simplified ecosystem of such agriculture is made possible by added chemical inputs, like fertilizers and pesticides, which have adverse environmental and human health effects. Research into alternative types of agriculture, like organic agriculture and agroecology, are needed to address the downsides of industrial agriculture. For example, several practices can be applied to manage pests (Table 1) [303]. They suggest that current yield gaps in organic systems compared with industrial systems reflect a research and development investment gap. Part of this gap is the use of biodiversity to diversify agriculture and develop cultivars adapted specifically to organic or other types of agriculture. This approach would maximize genotype by environment interactions to develop cultivars specifically adapted to organic environments ([304–306]; See also The use of biodiversity in genetic improvement).

## The use of biodiversity in genetic improvement

### The success of plant breeding

The second central avenue for agricultural biodiversity use to improve production, quantitatively and qualitatively, and the food system, more generally, is genetic improvement or plant breeding, i.e. the development of improved breeding lines or varieties (G factor: [257]). There can be no doubt that genetic improvement has been very successful, with steady increases in yield over the last decades or century (e.g. [307–309]), although there are significant differences among crops and environments. Major crops like main cereals (maize, rice) have benefited from the research and development investment. In contrast, grain legumes and other understudied crops have seen more limited progress (e.g. sub-Saharan Africa [308]). Also, the benefit of a breeding program depends on the relative emphasis on broad vs. narrow adaptation. In maize trials planted from 1999–2018, a large «home field advantage,» accounting for 19% of G × E or ∼8% of the mean yield was observed [310]. Location was responsible for 45% of yield variation, suggesting that selection for local adaptation could bring additional yield increases.

While this measurement of return on investment focuses mainly on yield, they do not reflect the negative environmental and social externalities arising from adopting improved varieties, such as those deriving from the Green Revolution [311,312]. Plant breeding needs to address these externalities, which are now known [313,314]. For example, the Green Revolution has caused a reduction in overall crop diversity because of its focus on wheat and rice. Rice-wheat cropping systems now dominate important food production areas worldwide [315]. In the Indo-Gangetic Plains and the Peninsular Region, 60% of the cropping area is under rice or wheat, replacing 25 different crops in the rainy and summer growing season. Continuous cultivation of cereals has led to a decline in soil organic matter and soil health. The introduction of short-cycle, high-value grain legumes like green or black gram could fit well into the rice-wheat cropping systems.

Thus, biodiversity plays a vital role in improving cropping systems. It is both a target to conserve through land-saving through increased productivity and a tool to achieve an environmentally more benign agriculture by introducing targeted traits into improved cultivars, like water or nitrogen efficiency [50,316,317].

### Two major trends in plant breeding reflected in the breeder's equation

Since its inception at the beginning of the 20th century, two major, linked trends have characterized plant breeding: (1) There is an increased emphasis on widening the types of biodiversity that are used in genetic improvement, and (2) There is a history of successive adoptions of new knowledge of plant genetics and physiology, technologies, and approaches that aim at increasing the efficiency of the selection process [318–321]. The two trends complement each other as the broadening of the genetic basis of crops is facilitated by new selection tools, and, conversely, the new tools can facilitate introgression or development of new traits. The process of genetic improvement can be conceptualized as a circular pipeline in which additional genetic diversity is introduced as needed by evolving objectives, and genetically improved materials from a previous breeding cycle serve as the raw material of the next breeding cycle. The throughput in this pipeline is governed by the so-called breeders’ equation [322,323], which states that response from selection (ΔR) is a function of the heritability (narrow– or broad-sense), the selection differential (S) and the duration of the cycle (t):

To increase *ΔR*, the central objective of genetic improvement, one can increase the heritability in the population or the selection differential and decrease the duration of the selection cycle. Increasing heritability can be achieved by increasing the total genetic or additive variances (see Sources of biodiversity for genetic improvement) or reducing the environmental/residual variance, which explains the importance of adding genetic diversity to breeding populations and increasing the precision of measurements on which selection is based. This will allow both an increase in selection intensity and selection accuracy [324]. Decreasing the duration of the cycle can be achieved, for example, by shortening the juvenile phase or speed breeding. Alternatively, the number of cycles/year can be increased in annual crops [325–329].

### Sources of biodiversity for genetic improvement

**Figure 5.**
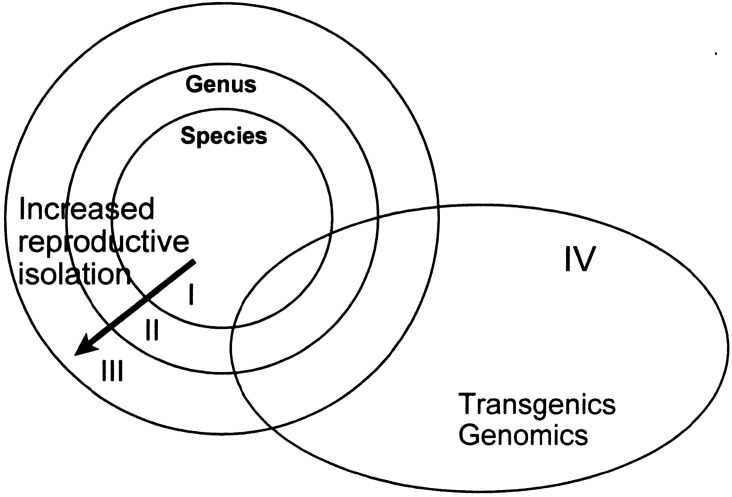
The concept of gene pools in plant breeding. This concept [188] reflects increasing reproductive isolation from Gene Pools I to III, and was extended by [335] to reflect the addition of genomic tools, transgenics, and gene editing, based on sequence information (Gene Pool IV). Permission requested.

Plant breeders can use a wide range of biodiversity sources. The choice of source will depend on the presence of the trait of interest and the ease with which the trait can be introgressed into the elite germplasm (see also Limitations and opportunities for gene transfer and introgression). Crop-related biodiversity can be categorized into three gene pools, from gene pool I to gene pool III, in increasing order of reproductive isolation [188]. Gene pool I coincides generally with the limits of biological species and includes wild ancestral and domesticated gene pools, given that these two gene pools are generally crossable without major viability and fertility issues. Therefore, there is extensive admixture between wild and domesticated types in centers of origin [170,191,330–334]. In contrast, gene pool III includes species that require extensive crossing attempts and special techniques like *in vitro* embryo culture. The progenies, if any, show limited viability and/or fertility. A fourth gene pool (Gene Pool IV; Figure 5) is added [335], which encompasses all other sources of biodiversity that are sexually incompatible. Adding gene pool IV considers the introduction of genes by transgenesis and gene editing but also information across species based on synteny and sequence homology.

**Figure 6.**
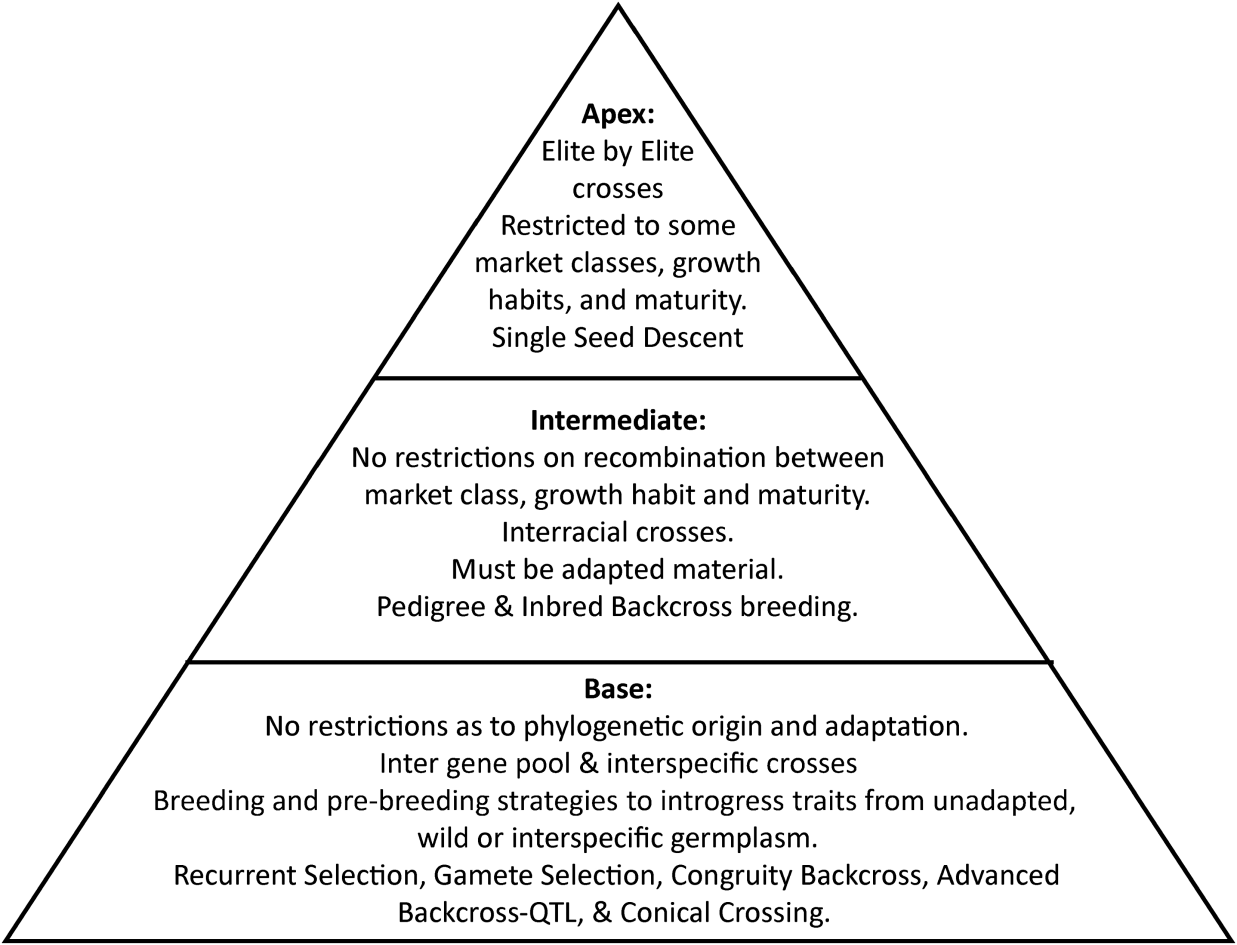
The breeding pyramid illustrating the utilization of various types of biodiversity. The introduction of biodiversity into elite, well-adapted varieties (top of the pyramid) relies on different breeding methods as a function of reproductive barriers and lack of adaptation to overcome. From [336], with permission.

Among the different sources of crop-related biodiversity, one can distinguish — in decreasing phenotypic relatedness — other elite cultivars, advanced breeding lines, obsolete cultivars, landraces or farmer varieties, ancestral wild relatives, and other species (primarily wild, but including domesticated types as well) [315,327]. Plant breeders understandably prefer biodiversity sources that are as closely related as possible to their elite materials to ease the recovery of desirable progeny, but — when necessary — more distant sources have to be used to satisfy the need for specific traits (Figure 6, from [336]).

There has been increasing attention paid to landraces and conspecific wild relatives as a source of biodiversity for a variety of traits (wild [327,337–339]; landraces: [340–346]. Traits from crop wild relatives included disease and pest resistances and abiotic stress tolerances, which has led to increased attention to these wild relatives (e.g. U.S.A. [347], Italy [348]).

In recent years, *de novo* domestication has been increasingly proposed to complement the existing crop portfolio. *De novo* domestication represents the initial domestication of a wild species that may or may not have been exploited (wild harvest) or cultivated (in a wild stage) before. Domesticating wild species reflects the introduction of essential domestication traits, which are part of the domestication syndrome [349,350]. This strategy is based on the fact that domesticated plant species only constitute a minor part of the plant kingdom. Various estimates limit the number of domesticated plant species to a few thousand, whereas the total number of plant species reaches some 400 000 species, seemingly providing an ample reservoir of additional potential domesticates. The domestication process can be accelerated by gene editing of the rapidly growing number of domestication genes that have been identified [44,156,351–353].

Early proposals of this approach are the potential *de novo* domestication of *Solanum galapagense*, a salt-tolerant relative of tomato (*S. lycopersicum*) [354] and other Solanaceae [355]. In an actual example four traits were introduced (compact plant type, day-length insensitivity, enlarged fruit, and increased vitamin C level [356]. Other examples of gene editing of putative domestication genes are provided in the groundcherry (*Physalis pruinosa*), a wild relative of the domesticated husk tomato (*Physalis philadelphica*), with characteristics similar to the wild tomato (*S. pimpinellifolium*) [357], and pennycress (*Thlaspi arvense*) [358]. Additional examples of the role of gene editing in domesticated plants include disease resistance in banana, cassava, maize, potato, rice, and wheat, food safety in cassava and rice, and insect resistance in rice [359].

The advantage of the genome editing approach to *de novo* domestication is the targeted, non-random modification of key genes that can achieve part of the phenotypic transition from a wild type to a domestication syndrome in a relatively short time (at least compared with the traditional domestication process). These key genes control developmental processes like seed dormancy, plant type, flowering time, and seed dispersal and often have a major effect [12,13,360]. Obtaining a domesticated version of these traits by gene editing is relatively easy but probably requires additional interventions by selective breeding to achieve the desired phenotype. Ultimately, yield is critical [361], and gene editing is a valuable tool among the many that plant breeders employ, but it is not a panacea [359,362,363].

An example is flowering time. A late-flowering wild plant can be transformed into an earlier-flowering plant by editing the photoperiod-sensitivity gene it carries into a photoperiod-neutral allele. This step will assure flowering, but further selections are needed to ensure early flowering imposed by the cropping systems in which this *de novo* domesticated plant will be cultivated. So, the success of *de novo* domestication — and the potential role of gene editing in this process — depends on the genetic architecture of the domestication syndrome (i.e. the number of genes, their magnitude, and their linkage relationships). Gene editing is handy if major genes are present; additional genes of smaller effect can then be further added by selective hybridization. The domestication syndrome likely is controlled by networks of genes that represent developmental, physiological, and metabolic pathways, which include different types of gene actions, including epistatic actions, and are now being discovered (e.g. [364–366]). Further conditions are the availability of transformation and regeneration systems for the target species. It is not a coincidence that many of the examples used the Solanaceae members, which are relatively easy to transform [367].

### Limitations and opportunities for gene transfer and introgression

Whether or not individual accessions of wild relatives or landraces of crops can provide the necessary traits depends on several factors that must be considered for a successful outcome of gene transfer into improved varieties in plant breeding programs.

Wild relatives and landraces are very variable; not every accession carries the trait of interest. Thus, biodiversity needs to be evaluated to identify those sources that carry the highest expression of the trait. This is generally feasible for disease and pest resistances but is more difficult for other traits, like yield, because of plant type and physiology differences. Wild accessions also have a stronger population structure and a narrower adaptation. Thus, direct evaluation of yield is generally not informative; an alternative is an indirect and more cumbersome evaluation in the progenies of crosses with a domesticated tester in early or later generations (e.g. common bean [368,369]).

Results of comparisons between wild and domesticated common bean and introgression of wild bean diversity into the domesticated gene pool [242] showed that a limited number of traits distinguish the drought response of wild and domesticated beans. The advantage of wild beans under drought stress is their continued growth and the reconfiguration of the root system towards less branching and deeper tap roots. In addition, field evaluations showed that only wild beans from arid environments, which have deeper root systems, contributed yield increases to domesticated × wild progenies under drought stress but not under well-watered conditions [369]. Thus, in this case, focused on drought tolerance, effective introgression depended on selecting the right accession (wild accessions from arid areas), measuring the right trait (continued growth and deeper root systems), and evaluating the progenies under the right conditions (field experiments under limited water supply).

A second condition is that the accessions can be crossed to targets to be improved (Gene pools I–III [188]). Alternatively, the genes must be isolated and transformed by transgenesis or edited based on pre-existing information (Gene pool IV, see Limitations and opportunities for gene transfer and introgression). This information is crucial for germplasm utilization and requires prior research on taxonomy and systematics, and reproductive isolation among the different taxa [370,371]. Further research is needed to overcome these reproductive isolation barriers.

For example, crosses between common bean and tepary bean (*Phaseolus acutifolius*, Gene pool III) can now be facilitated through crossing of common × tepary bean lines with a wild relative of tepary bean (*P. parvifolius*), to increase male gamete diversity [372]. Progeny lines of the common × tepary × parvifolius had large chromosome segments inherited from *P. parvifolius*; when crossed with tepary beans, the progenies had significantly improved viability and fertility. This approach opens up the possibility of transferring quantitative traits related to abiotic and biotic stresses associated with climate change between the two domesticated *Phaseolus* species, such as heat and drought tolerance of tepary bean native to the arid southwest U.S. and northwest Mexico.

Nevertheless, recombination remains an overarching concern in introgression and general breeding. The number of cross-overs in normal meiosis is approximately two per bivalent or one per chromosome arm. This is the main constraint in plant breeding, especially among phylogenetically more distant taxa [373]. In a mutagenesis experiment of rice, pea, and tomato orthologues of recombination-rate-modifier genes of Arabidopsis, a mutation in the RECQ4 gene increased recombination three-fold in these crops [374]. It could be used more generally in breeding, especially in introgression combined with speed breeding, genome editing, and genomic selection (see below in this Section).

A third condition is that the appropriate breeding methods can be applied to transfer traits from exotic, unadapted accessions into elite backgrounds. Such procedures must consider the genetic architecture of the traits, any reproductive isolation, the reproductive system of the crop, and the type of cultivar, whether a population, F_1_ hybrid, or pure line. This phase is usually called pre-breeding as it is intermediate between the germplasm, unimproved status, and the elite, improved status. It is, however, a critical step in utilizing gene bank entries because it transfers traits of interest into an adapted background (e.g. [375–377]).

There are several breeding tools available to bridge the adaptation gap, such as. various types of backcrossing (e.g. [369,378,379]). Another approach is germplasm conversion, in which the introgression process is turned on its head. Instead of introgressing a trait of interest into an adapted background, genes for adaptation (e.g. photoperiod neutrality, plant type, seed color) are introgressed into unadapted accessions). This approach has been put into practice in sorghum, where photoperiod-insensitive, short-stature progenies were selected [380]. Genomic tools allowed to follow the introgression of two linked, recessive genes on chromosome SBI-06: the early flowering allele with the major effect, recessive *ma1*, among six *Ma* loci, and the dwarfing allele *dw2*, among four *Dw* loci [381].

Other breeding approaches can be used, especially if they maintain heterozygosity to promote effective recombination. These include congruity backcrossing [382], inbred backcross [383–385], advanced-backcross QTL [386,387], and conical crossing or Multi-Parent Advanced Generation Inter-Cross (MAGIC) populations [388–390].

### Combining genotypic, phenotypic data, and ecogeographic data of crop-related biodiversity

These populations’ development is combined with applied genomic tools, including genome sequencing and re-sequencing, gene editing, genome-wide association and quantitative trait loci studies (GWAS and QTL [391]). The combination of population development and the application of genomics-assisted breeding (GAB) is a powerful approach to characterize and manipulate allelic variation, create novel genetic diversity combinations, and promote their more efficient incorporation into elite improved gene pools [392]. A considerable effort goes into the genotypic characterization of gene bank collections by re-sequencing large samples of these collections (e.g. rice [393,394]; molecular passport data [395]; and pan-genomes [396–400].

A key aspect is the combination of these genotypic data with phenotypic measurements or observations. A GWAS approach, combined with participatory approaches and quantitative genetics, was used to understand the genetic basis of durum wheat quality preferences by smallholder farmers in the Ethiopian highlands [401]. Farmers contributed quantitative evaluations of agronomic and quality traits of 400 local wheat landraces, which were genotyped with SNPs. The farmer evaluations were partially influenced by gender and were repeatable and heritable. The GWA study showed that smallholder farmers’ traditional knowledge could yield QTLs, partially dependent on gender and location. Traditional knowledge should be incorporated into crop breeding to obtain better locally adapted cultivars.

The concept of genomic prediction is now being extended from breeding populations to the holdings of gene banks. The genome prediction strategy was applied with a training set of 299 accessions selected from an overall set of 962 biomass sorghum accessions [402]. Cross-validation of the training set biomass data indicated moderate to high prediction accuracy (ranging from *r* = 0.35 to 0.78 across different traits). In a 200-accession validation subset, the authors obtained a high prediction accuracy (*r* = 0.76). This study suggested that this approach could be extended to other crops and traits in other gene banks as well (e.g. [403]).

With sequencing becoming ever cheaper, more efficient, and more accurate, the limiting factor becomes high-throughput plant phenotyping. This is especially the case in gene banks, where the number of accessions may reach thousands. To resolve this bottleneck, multiple proximal remote sensing technologies have been proposed [404]. These include various types of imaging, including RGB, thermal, hyperspectral, and fluorescence imaging, which are non-destructive and offer a more rapid data collection compared with traditional phenotyping methods. Both indoor and outdoor platforms are available [405–408]. Research is needed to determine the full range of traits that can be measured and their relation to crop performance with these high-throughput approaches.

To this end, phenotypic results can be combined with genotypic and modeling results to identify candidate genes or genome regions of interest [406,409].For example, a pilotless aerial vehicle (drone) was used to quantify early-development growth vigor, a trait involved in weed control and reduced water consumption, in a recombinant inbred population of common bean resulting from the cross between a slow- and fast-growing variety of common bean [406]. This procedure enabled an efficient monitoring of the growth rate of individual progeny lines and conducting a QTL analysis, which showed that early vigor was genetically controlled and was the result of complex interactions between several QTLs and environmental factors. Remote sensing enables a high-throughput quantification of growth rate and physiological traits (e.g. leaf temperature) and should be applied to gene bank collections. An analysis of a maize half-diallel planted in multiple locations of the U.S. Corn Belt representing a range of water availability, showed that the combination of crop growth models and genomic prediction provided an enhanced predictive accuracy and could identify candidate physiological traits known to explain variation in maize yield [409].

More generally, there is a need to integrate gene bank data with environmental data about the location of collections *in situ*. At the very least, these locations should have geographic coordinates (georeferenced). Still, other data such as vegetation and soil types, disease and pest symptoms, and, if possible, traditional knowledge should also be included. Databases that combine all these different types of data, including phenotypic, genotypic, environmental, and ecological data, should be part of a vision of future gene banks. The Genesys-PGR database groups traditional gene bank data (accession and passport data) for over 500 collections worldwide. It contains over 4 million gene bank accession data, over half of the 7 million conserved globally ([410]; https://www.genesys-pgr.org/). Ancillary databases are Plant JSTOR (https://plants.jstor.org), the Global Biodiversity Information Facility (https://www.gbif.org), and EURISCO (https://www.ecpgr.cgiar.org/). More specialized environmental databases are WordClim (https://www.worldclim.org/), Eto Calculator (https://www.fao.org/land-water/databases-and-software/eto-calculator/en/), and ISRIC (World Soil Information).

## Legal, policy, and valuation framework

### National intellectual property rights and international treaty regimes

Since 1980, the international and national legal landscape in which biodiversity is considered has changed markedly [125]. Before that year, biodiversity was considered the «common heritage of humanity» [411]. Under this concept, biological resources were treated as belonging to the public domain and could not be owned by any individual, group, or state. In 1980, the U.S. Supreme Court overturned a U.S. Patent Office decision that had rejected an application for a patent covering a novel, genetically engineered bacterial strain capable of degrading hydrocarbons [412]. This decision set in motion subsequent decisions of the court allowing patents on novel genes and organisms, including crop cultivars, facilitated by the advent of molecular genetics. The possibility of obtaining a utility patent to establish an intellectual property right on a cultivar joined the earlier legal protections on cultivars, namely the plant patent and plant variety protection (PVP) [125,413]. Obtaining a utility patent for a cultivar became the preferred intellectual property right (IPR) because, unlike PVP, it had no farmers’ or breeders’ exemptions. These exemptions allowed farmers to re-use harvested seeds of a PVP cultivar and breeders to use the same as a parent in a breeding program. Given the perceived importance of patents in stimulating economic activity, other countries followed suit. They established their own cultivar or gene intellectual property rules, notably in the European Union and Japan.

In parallel with these changes at the national level, the international scene changed as well, mainly through the implementation of treaties [125], the most important of which is the Convention on Biological Diversity (CBD, 1993), the Trade-Related Aspects of Intellectual Property Right (TRIPs, 1995), and the International Treaty on Plant Genetic Resources for Food and Agriculture (the «Plant Treaty,» 2004). The CBD specifies that ownership of and control over biodiversity resides with the states and that there should be fair and equitable access and benefit sharing (ABS) regarding biodiversity. The TRIPS provision imposed on countries who wanted to join the World Trade Organization the obligation to institute an IPR regime in their respective countries, covering biodiversity, except soil microbes. The Plant Treaty is a multi-lateral agreement governing the free exchange of a specified list of ∼100 crops. It provides hope that more flexible biodiversity exchange regimes can be established, provided that individual countries are willing to abide by the specifications of the treaty.

The main goals of the IPR legislations and treaties are to stimulate the fair use and efficient conservation of biodiversity. Have these goals been achieved? The answer is generally no. Functions and Uses of Biodiversity and Trends in crop biodiversity detail the overall biodiversity losses due to several causes, including climate change and habitat loss. Furthermore, the legislation and treaties have drawbacks limiting their potential benefits. In mining patent documents,it can be shown that patent activity only concerned 4% of taxonomically described species or ∼1% of predicted species [414]. Activities involved a limited scope of pharmaceuticals, traditional medicine, foods, biocides, marine genetic resources, and Antarctica. The authors suggested that a broader spectrum of biodiversity needs to be opened up based on the principles of ABS and other objectives of the CBD. They also argue that alternative innovation approaches, such as open source and commons models, should be instituted to involve biodiversity in research and development in areas of human needs. Regarding cultivar development specifically, alternatives to the strong property paradigm represented by the patenting and PVP regimes could be pursued, including stronger farmers’, breeders’, and research exemptions [413].

### The Kunming-Montréal global biodiversity framework (GBF)

Faced with the twin crises of climate change and biocultural diversity loss, an ambitious framework (officially called the Kunming-Montreal Global Biodiversity Framework; https://www.cbd.int/article/cop15-final-text-kunming-montreal-gbf-221222) was agreed upon at the 15th Conference of the Parties to the CBD (December 2022), which set up targets and goals to restore, maintain, enhance, and sustainably use biodiversity across the world by 2030 and 2050, respectively [415–418]:

- Among the targets is the sustainable use of biodiversity in areas under agriculture and forestry through agroecological approaches, maintaining ecosystem functions and services, and linking biodiversity conservation with people.
- Also, the GBF targets the establishment of an equitably-governed system of protected areas, recognizing indigenous and traditional territories and practices and leading to effective conservation and management of at least 30% of terrestrial and marine areas, whereas, currently, only 17% and 10%, respectively, of these areas are protected.
- Make available at least U.S. $200 billion per year in biodiversity-targeted funding, by 2030.

The GBF seeks to go beyond the mere enumeration of causes of biodiversity loss. It proposes interventions to halt the main biodiversity loss factors and protect and restore biodiversity in conjunction with effective actions to mitigate climate change. A potential example of the application of the GBF is the conservation of *Phaseolus* germplasm from wild to landraces to improved varieties (Table 2) [419].

**Table 2:** Factors affecting genetic vulnerability and resilience/conservation of *Phaseolus* beans biodiversity ([419]: their Table 8, with permission)

Feature or crop evolutionary stage	Genetic vulnerability	Genetic resilience/conservation
Centers of origins (primary centers of diversity): wild populations	Habitat destruction through expansion of urban areas, industrial agriculture, and non-agricultural activities;	Natural areas or biosphere reserves
	Global warming leading to extreme weather events (drought, hurricanes);	Land abandonment
	Political instability;	Ecogeographic model of explorations
	Slowdown in explorations	
Traditional and indigenous bean production systems: domesticated populations, i.e. landraces	Gene flow from wild to domesticated populations;	Gene flow from wild to domesticated populations;
	Human migration from rural to urban areas on domesticated populations; land abandonment	Introduction of additional cultivars through local seed systems;
	Political instability;	Creolization;
	Lack of conservation of seeds of released varieties	Traditional knowledge about adaptation and varietal traits;
		Adoption of different Phaseolus species
Secondary centers of diversity: domesticates	Selection for adaptation and different uses;	Hybridization among varieties and gene pools;
	Genetic bottlenecks	Adoption of different Phaseolus species
Conservation and breeding	Limited systematic genotyping and phenotyping of gene bank collections;	Large gene collections in multiple gene banks
	Repeated use of same sources or genes to address breeding objectives	Diversity panels;
		Whole-genome sequencing and reference sequence for the two gene pools and selected ecogeographic races;
		Search for alternative sources of resistance or tolerance;
		Pyramiding genes;
		Hybridizations among gene pools and ecogeographic races
		Genomics-assisted selection strategies
Large-scale, industrial production	Single-genotype fields;	Growing regions including different gene pools and ecogeographic races
	Similar pedigrees	Geographic distances among growing regions

### The Nagoya protocol and digital sequence information

The ABS goal of the CBD has been one of the most challenging provisions to implement [420]. This goal was meant to counteract the unilateral exploitation (if not biopiracy) of biodiversity and support the cost of conservation and socio-economic development of biodiverse countries of the Global South by sharing benefits arising from the use of biodiversity with the original suppliers of the biological materials through bilateral agreements between suppliers and users. To clarify the ABS goal, the Nagoya Protocol (NP) on Access to Genetic Resources and the Fair and Equitable Sharing of Benefits Arising from their Utilization was implemented in 2014 (https://www.cbd.int/abs/).

Several difficulties exist with the ABS goal, however, which include (1) the vagueness of the original wording of the CBD regarding the concept of utilization and its temporal scope; (2) the lack of recognition of non-commercial and public domain research and the need for unencumbered access to biodiversity samples and specimens for non-commercial research; (3) the lack of intrinsic research or market value of biodiversity that would lead to direct commercialization without research to generate knowledge that may lead to innovations downstream; (4) the substitution of patents by trademark or copyright provisions to maintain inventions under private control and outside national compliance measures of the Nagoya protocol; (5) anticipated benefits from the commercial use of biodiversity, which might have been directed to local or indigenous people, have not yet been realized. This situation has led to a decline in local research by in-country researchers, international research collaborations, and the use of biodiversity from *in situ* sources, along with training and technology transfer leading to capacity building. Publicly funded, non-commercial research has come under scrutiny, including through freedom-of-information or public-records demands, while private, commercial research remains unaffected by ABS provisions [420,421]. The reduction in the number of explorations for *Phaseolus* germplasm in Latin America in the last decades [419] can be attributed at least in part to increasingly restrictive sovereignty and intellectual property regimes operating that have been put into force in the last three decades.

An additional issue is the nature of biodiversity concerning research and development. Biodiversity utilization in, for example, bioprospecting and plant breeding, has shifted — at least partially — away from physical resources such as planting materials like seeds and towards information, such as Digital Sequence Information (DSI; see also Sources of biodiversity for genetic improvement: Gene Pool IV) [422]. The importance of DSI in our understanding of biodiversity, its diversity, its functional characteristics, and its molecular and biochemical basis cannot be overestimated. Because of its importance, DSI has become an object of contention in the discussions at the international level about ABS.

There is a lack of a clear definition of DSI under the CBD, NP, and Plant treaties [423]. Because information is non-physical or dematerialized, unlike seeds or other planting materials, it can be replicated without physical access [424]. Thus, DSI can theoretically undermine ABS [423]. Nevertheless, an important caveat is that the mere availability of nucleic acid sequences is insufficient to specify individual traits, let alone an entire organism. Numerous expression steps are required between the genotypic information (G) provided by a DNA sequence and the organismal phenotype. In addition, environmental effects (E) and G × E interactions should also be considered to account fully for biodiversity complexity. Thus, DSI, extracted from their physical propagation structure (seed or other propagation materials), may be even more removed from the needs of farmers and the actual circumstances under which they operate [186,425].

An international treaty solution for DSI remains to be implemented, which will probably be separate from existing treaties, as it will probably be multi-lateral instead of bilateral [426]. However, there is now general agreement that the international community needs to find a mechanism through which countries can benefit fairly from the use of DSI and that the bilateral contract model of the CBD and NP is inadequate for this [426–428]. Particularly striking is the realization of the mutual benefit between DSI, on the one hand, and other types of information and materials (see Combining genotypic, phenotypic data, and ecogeographic data of crop-related biodiversity), on the other hand, including phenotypic data, biochemical and metabolomic profiles, spatial information, indigenous knowledge, and physical specimens [424].

## Summary

- Biodiversity, coupled with the accompanying cultural knowledge, is the premier environmental resource on Earth. The loss of biocultural diversity is unique among environmental threats in that it is irreplaceable and irreversible. As the general biodiversity goes, so does agrobiodiversity.
- Biocultural diversity provides multiple advantages, which include increased and more resilient and sustainable productivity, ecosystem services, socio-economic benefits and traditional knowledge, and diet diversity.
- Several anthropogenic factors have reduced biocultural diversity, including agriculture and domestication, human ventures and crop dissemination, migration and land abandonment, evolving economic ventures and seed systems, social and gender barriers, and contemporary agricultural and food systems.
- Cropping improvement (the practices of growing plants) can improve biodiversity through an increase in diversity at the field, agroecosystem, and landscape levels, by incorporating diverse cultivars, alternative and understudied crops, *de novo* domestication, and functionally diverse agricultural landscapes.
- Genetic improvement can enhance biodiversity by broadening the sources of diversity available *in situ* (farmers’ fields and natural areas) and *ex situ* (gene banks) to include crop wild relatives and other species and utilizing the steady progress in novel breeding approaches, including genomics in all its facets, bioinformatics, speed breeding, and genetic advances.
- Biocultural diversity has been included in national legal frameworks regarding intellectual property and international treaty frameworks aiming to conserve biodiversity but also assure its access and the sharing of benefits (ABS) resulting from its use. The Kunming-Montréal Global Biodiversity Framework provides goals and targets to conserve and restore biodiversity across the world. Access and benefit sharing remains a point of contention, mainly as it now includes digital sequence information (DSI). However, an equitable solution to the ABS and DSI issues may yet provide a solution for the sustainable use of biocultural diversity.

## Open Access

Open access for this article was enabled by the participation of University of California in an all-inclusive *Read & Publish* agreement with Portland Press and the Biochemical Society under a transformative agreement with UC.

## Abbreviations

ABS: access and benefit sharing
AHZ: Amotape-Huancabamba Zone
DSI: Digital Sequence Information
IPR: intellectual property right
NGRAC: National Genetic Resources Advisory Council
NP: Nagoya Protocol
PVP: plant variety protection
SNPs: single-nucleotide polymorphisms
VCM: vegetation-climate mismatch
WGD: whole-genome duplications
